# Social networks and inference about unknown events: A case of the match between Google’s AlphaGo and Sedol Lee

**DOI:** 10.1371/journal.pone.0171472

**Published:** 2017-02-21

**Authors:** Jonghoon Bae, Young-Jae Cha, Hyungsuk Lee, Boyun Lee, Sojung Baek, Semin Choi, Dayk Jang

**Affiliations:** 1 Graduate School of Business, Seoul National University, Seoul, Korea; 2 Transdisciplinary Research Center for Culture-Brain Dynamics, Seoul National University, Seoul, Korea; 3 Interdisciplinary Program in Cognitive Science, Seoul National University, Seoul, Korea; 4 Interdisciplinary Program in History and Philosophy of Science, Seoul National University, Seoul, Korea; 5 Department of Statistics, Seoul National University, Seoul, Korea; 6 College of Liberal Studies, Seoul National University, Seoul, Korea; Centre de physique theorique, FRANCE

## Abstract

This study examines whether the way that a person makes inferences about unknown events is associated with his or her social relations, more precisely, those characterized by ego network density that reflects the structure of a person’s immediate social relation. From the analysis of individual predictions over the Go match between AlphaGo and Sedol Lee in March 2016 in Seoul, Korea, this study shows that the low-density group scored higher than the high-density group in the accuracy of the prediction over a future state of a social event, i.e., the outcome of the first game. We corroborated this finding with three replication tests that asked the participants to predict the following: film awards, President Park’s impeachment in Korea, and the counterfactual assessment of the US presidential election. Taken together, this study suggests that network density is negatively associated with vision advantage, i.e., the ability to discover and forecast an unknown aspect of a social event.

## Introduction

People predict. Presidential candidates seek to identify what the majority of the voters want the most; marketing managers try to predict consumers’ preference on a newly launched product. It is an essential aspect of human sociality to make inferences about unknown states of a social phenomenon. A social event takes different states, depending on the efforts and capabilities of individuals that are associated with the event. Because the efforts and capabilities of the others are not under the direct control of the observer, the evolution of a social event is typically unknown to the observer. For example, the popularity of a new song, the capability of a new hire, and the result of the upcoming election are indeterminate, unknown states of musical tastes, labor productivity, and political orientations, respectively.

A challenge for the inference about the social world is that information about the unknown states is not readily available. Rather, social relations serve as a conduit for and thus an obstacle to the availability of information about the social world. Those rich in social relations would more easily guess the unknown states of the world. Indeed, the sampling model of judgment and decision-making posits that a person’s judgement hinges upon his or her knowledge of the social world. The knowledge of the social world would vary with the information that the person obtains from available samples, i.e., social contacts in the external environment [1,2]. Despite a vast body of research done on social relations [3,4,5], little is known about the role of an individual’s social relations in guessing the unknown states of the social world, namely, vision advantage [3].

Accuracy in guessing, i.e., the error in inference, is a function of both the bias and the variance of sampled predictions. Given that social relations serve as a conduit for the samples of the others’ experience and knowledge [3,6], the accuracy of guessing would then increase as a large number of social contacts are independently drawn from the social world. However, a person’s immediate social relations are remote from this requirement. The samples of the others’ knowledge of the social world are prohibitively small because there certainly is a limit to the number of social contacts that an individual maintains in a meaningful manner [7]. Even worse, such samples obtained from social contacts are biased due to homophily in social relations [8,9]. Prior to the formation of social relations, individuals of similar opinions may get attracted to each other and develop social relations among themselves. Opinions in an individual’s social relations are not independent.

This limitation draws the attention of researchers, mostly sociologists, to the pattern of social relations such that mutually disconnected social contacts may yield non-redundant samples of knowledge as to the social world [3,10,11,12]. To the extent that mutually disconnected contacts have independent, conflicting preferences and experiences, the samples of knowledge that a person obtains from her mutually disconnected contacts are likely to reflect different views as to the world. Moreover, such disconnected contacts may alleviate a person’s conformity tendency, which may impair the accuracy of guessing about unknown states of the social world [13,14]. The person thus tends to make an informed and independent judgment about the social world. As is the case with the prediction market or simply the wisdom of the crowd effect [15], diverse knowledge as well as independent decision may help the individual to improve the accuracy of her guessing as to the unknown social world.

**Fig 1:**
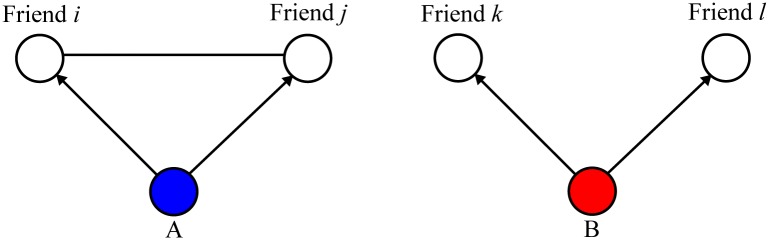
Types of social capital: An ego-network level.

But again the same pitfall may apply to this prediction. Given the restrictive number of social contacts a person has, the value of diverse knowledge may be still limited. Indeed, another group of researchers suggest that mutually connected contacts, namely transitive relations, may generate more information than their disconnected counterparts do [16,17,18]. Suppose that person *A* has redundant contacts and person *B* has non-redundant contacts (Fig 1). This indicates that person *A* belongs to closely knit social relations, whereas person *B* bridges two disconnected social groups. It is likely that mutually disconnected social contacts serve as the source of knowledge diversity, which is critical to the accuracy of estimates about the unknown states of the social world [3,15]. In comparison, it is also likely that the friends of person *A* in Fig 1 are more cooperative, i.e., more motivated to share their opinions with person *A*, whereas those of person *B* would rather be reluctant to share what they know with person *B*. This observation rests on the following two mechanisms. One is that the motivation to share knowledge increases as people belong to a closely knit social circle [19,20,21,22]. Whether a person’s social contacts are mutually connected or not is one indicator to such social cohesion. The other is more about the decision-making style of a person [23,24,25,26,27]. It states that the person of the cohesive social group not only conforms to the opinions of the other members, but knows what the others expect of the person. The person is capable of reading the mind of the others.

This discussion is well reflected into the following two distinct, sociological models of social relations: network brokerage and network closure [3,18,26,27]. These models serve to evaluate the structure of social relations that surround a focal person, namely, an ego network. They draw on the neighborhood of a person on a social network and thus utilize information only on a local structure of a global network. From the perspective of a focal person, the network brokerage model describes the social and economic benefits of a low-density ego network that is rich in non-redundant contacts (Person *B* of Fig 1). In comparison, the network closure model depicts the benefits of a high-density network that is lack of non-redundant ones (Person *A* of Fig 1). Insofar as a person’s social relations limit his or her understanding of the social environment, a person’s social inference should vary with social relations in his or her ego network.

These models have developed in isolation of models of global network or network topology [28,29]. One notable exception is a study on the strength of weak ties [10], which motivates models of small-world and scale-free networks [30,31,32]. A common observation behind the models of global network is that the properties of global network such as the average shortest path length cannot be reducible to those of local network such as ego network density [33,34,35]. An emphasis is accordingly given to uncovering the structure of global network.

The models of social influence at the global network level present two distinct channels for opinion formation: the active role of super-connectors or the hubs and the impact of densely-interacting social circles. The former emphasizes the advantage of the hubs to expose a common idea to non-interacting persons [36,37], whereas the latter focuses on the importance of a person’s multiple exposures to a common idea [22,38,39]. In a similar vein, the models at the ego network level suggest that two distinct channels are present, i.e., non-redundant and redundant contacts, each of which may correspond to the hubs and the circles.

However, ego network itself is a distinct building block for a global network. Its properties are not necessarily correlated with those of a global network. It presents the immediate social environment that constrains and supports the decision-making of each person [40,41]. The everyday experience of a person is subjectively shaped within this immediate social environment. Sociological models of ego networks are thus to assess the role of the immediate social relations in shaping a person’s decision-making, i.e., social inference.

Social inference is an mirror image of social influence and is about personal understanding of the social world [42,43]. In comparison, social influence by others underlies the formation of consensus via social relations [36,37,38,44,45]. Models of social influence thus seek to examine whether social relations inhibit independent decision-making and thus impair decision quality at the group level [13,14]. Insofar as individuals are already embedded in social relations, however, it would be more realistic to ask which type of social relations would impair or improve decision quality. The models of ego network, i.e., network brokerage and closure, seek to address this question with respect to social inference about uncertain future events, which is dubbed as vision advantage [3]. Accordingly, the focus of this study is to examine the individual variation in opinions, not consensus-building. In other words, we seek to identify an individual whose ideation is better at the ego network level.

Various tests have been made to measure indirectly behavioral outcomes such as economic achievement and personal rewards that reflect the interplay of social relations and the individual variation in decision-making [46]. The empirical tests of creativity, innovation outputs [23,47,48,49] and job search [11] are representative. Nonetheless, little attempt is made to assess directly the variation in decisions, in particular, vision advantage that arises from a person’s immediate social relations. A few direct tests concern only the virtue of relation-free guessing in such a domain as political election [50,51].

Against this backdrop, in this study, we examine whether a person’s inference about the unknown states of the social world may vary with the structure of his or her social relations. A test of vision advantage requires the prediction of uncertain social events whose outcomes are indeterminate at the time of prediction. Our experimental task was to predict a future state of a social event whose evolution was unknown to society as a whole. It differs from conventional estimation tasks where participants were asked to answer an ambiguous question whose solution was known to experimenters and was not necessarily available to participants [14]. To this end, we ran an experiment in which the participants were asked to predict match outcomes in the Go game between AlphaGo and Sedol Lee in March 2016 in Seoul, Korea and compared the individual variation in outcome predictions against the type of social relations that each person maintained. Moreover, we corroborated our finding with three replication tests in such domains as artworks and politics.

## Materials and methods

### Participants

**Fig 2:**
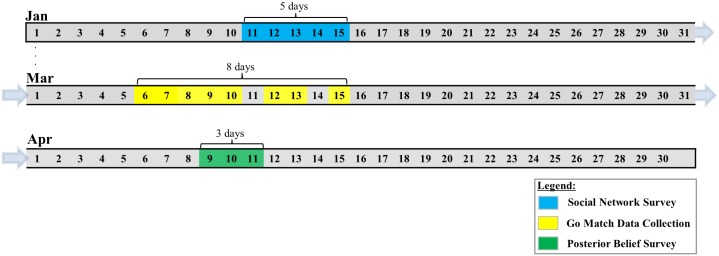
The schedule for three surveys. (A) Social Network Survey—offline (experimental room) (B) Go Match Data Collection—online; knowledge of Go & AI—offline (experimental room) (C) Posterior Belief Survey—offline (take-home); IRI Survey—offline (experimental room)

All participants were recruited from a long-term subject pool of the Culture-Brain Dynamics Transdisciplinary Research Center at Seoul National University, who agreed to participate in multiple experiments such as fMRI imaging and behavioral games during the academic year 2015–2016. One hundred undergraduate students enrolled in Seoul National University were independently recruited via Sona System, an online platform for lab management (https://snucube.sona-systems.com). Written informed consent was obtained from each participant and the surveys were administered in compliance with the safety guidelines for human experimental research, as approved by the Institutional Review Board at Seoul National University. Several days prior to each experiment, individuals in the pool received an e-mail that informed them of an upcoming experiment for a reasonable hourly wage. There were three different surveys in order (Fig 2 and S2 Text). The first survey was scheduled in January to measure each participant’s social relations (*N* = 76). The second survey was scheduled in March to measure each participant’s prediction about the Go-match between AlphaGo and Sedol Lee (*N* = 63). The third survey was scheduled in April to measure the ex post attitude of each participant about the outcome of the Go-match (*N* = 56). Owing to missing values on social relations, the final sample for the Go-match analysis included 43 observations, whereas that for ex post attitude in April included 37 observations. The data used for this study are available as S1 Dataset.

### Experimental procedures

First, from the 11^th^ of January 2016 to the 15^th^ of January 2016, a social network survey was conducted in a quiet room where the experimenter explained directly to the participants about the purpose of the survey and how to answer each question. Second, data for the Go-match were obtained via the short message service (SMS) of a phone from the 8^th^ of March to the 15^th^ of March 2016. An offline survey was also administered to measure each participant’s knowledge of Go and AI from the 6^th^ of March to the 8^th^ of March. Individual predictions of the first game outcome were measured from the 8^th^ of March to the 9^th^ of March. Four participants returned their predictions later than 1:00 pm when the first game began and earlier than 4:30 pm when the first game ended. For the remaining games, the predictions for the next game were collected when the previous game ended. Each participant received a google-form link via SMS, which displayed the questionnaires that assessed each respondent’s media exposure as well as emotional response to the result of the game. Lastly, those who participated in the Go-match survey were re-contacted and asked to return an envelope of questionnaires on their posterior belief in Lee’s capability as well as attributional styles from 9^th^ to 11^th^ of April, one month after the first game of the Go-match. When they returned questionnaires to the experimenter, they were also asked to complete a questionnaire on the Interpersonal Reactivity Index in an isolated experimental room.

### Social networks

For the identification of each participant’s social relations, we opted for so-called name generator and interpreter, a set of open-ended questions widely used in social networks research [36,52,53]. In particular, each participant was asked to name friends or mentors at school, i.e., direct contacts from whom he or she took advice on activities at school. In particular, they were asked for the first names or initials of a person “to whom you normally go for help and advice on work-related topics”. The social focus of our survey [54] was therefore task-based networks at school. Given that each participant was keenly concerned with the privacy of his or her social relations, we asked each participant to name as many direct contacts by citing either first names (not full names) or initials. We then asked each participant to report whether the direct contacts also knew each other, i.e., indirect ties. This procedure led to the identification of each participant’s ego network, i.e., the sum of his or her direct and indirect ties. A rationale behind this procedure is as follows. Social relations that are cited may be salient and important ones to respondents [55,56]. Accordingly, trivial ones that are not active may not be represented in this procedure. The survey took less than 10 minutes.

We employed ego network density to characterize a person’s social relations. It is the actual number of indirect ties in a person’s ego network, divided by the maximum possible number of indirect ties. It varies from zero to one. A high density indicates a closed group of friends where the focal person’s friends are friends by themselves [18]. In contrast, a low density indicates a broker’s network such that the friends of the focal person do not know each other [5]. For a total of 43 participants, the mean of ego network density was 0.49 (s.d. = 0.27). Ego network density in the sample was negatively associated with network size, i.e., the number of friends that each participant maintained (*r* = -0.44, *p* = 0.004). The mean of degree centrality was 5.65 (s.d. = 2.37), ranging from 2 to 13 (S2 Text).

### Social cognition

In one month after the Go match, we characterized each participant’s social cognition styles by assessing attribution styles and empathy with others, both of which may reflect a person’s way of processing events about and opinions of the others. For attributional styles, we opted for the Internal, Personal and Situational Attributions Questionnaire (IPSAQ) [57], where 32 items were presented as to positive and negative events. In particular, we examined the following two types of biases. One is externalizing bias, which was the number of internal attributions for positive events minus that of internal attributions for negative events. The other is personalizing bias, which was the number of personal attributions for negative events that was divided by the sum of both personal and situational attributions for negative events.

Moreover, we evaluated each participant’s way of interacting with others by using the Interpersonal Reactivity Index (IRI) [58], which measured the extent of a person’s empathy with others. This self-report index consisted of four subscales, i.e., fantasy, perspective taking, empathic concern, and personal distress. The content of each scale was the following [58]: the Fantasy scale reflected a person’s projecting oneself into the feelings and actions of fictitious characters in books and movies; the Perspective Taking scale was about a person’s propensity to adopt the perspective of others; the Empathic Concern scale indicated a person’s concern for unfortunate others; and the Personal Distress scale captured self-oriented feelings in tense interpersonal settings.

### Prior and posterior beliefs

We identified each participant’s belief in the Go capability of Sedol Lee to win against AlphaGo before and after the whole match in March 2016, Seoul, Korea. Prior to the match, the participants were asked to evaluate Lee’s capability on the following 6-point Likert scale item: ‘Sedol Lee, Go player of 9 dan rank, and AlphaGo, an AI program for Go game, will have a match that consists of 5 games. What is your prospect for the match?’ (5) Lee will win all of the games; (4) Lee will win most of the games; (3) Lee will win more games than AlphaGo; (2) AlphaGo will win more games than Lee; (1) AlphaGo will win most of the games; and (0) AlphaGo will win all of the games. In our analysis, those whose answer was either (5) or (4) were considered to have a high prior belief in the Go capability of Lee.

In April, one month after the match, the same participants were asked to evaluate Lee’s capability on the following 5-point Likert scale item that was reversely coded: ‘What do you think of the Go capability of Lee relative to AlphaGo?’ (1) AlphaGo is highly inferior to Sedol Lee; (2) AlphaGo is slightly inferior to Sedol Lee; (3) AlphaGo is as competent as Sedol Lee; (4) AlphaGo is slightly superior to Sedol Lee; and (5) AlphaGo is highly superior to Sedol Lee. In the subsequent analysis, those whose answer was either (1) and (2) were considered to have a high posterior belief in the Go-capability of Lee.

## Results

### Variation in priors

Before examining the role of a person’s social relations in making inference about uncertain events, we first identified a general pattern, if any, in each person’s inference about the outcome of the Go-match between AlphaGo and Sedol Lee. This observed pattern would be used as a baseline model for us to examine the role of social relations in making inferences. On the basis of each person’s prior belief over the Go-capability of Sedol Lee vis-à-vis AlphaGo, we split the participants into two groups, i.e., high-prior and low-prior groups. The high-prior group includes individuals whose prior beliefs or initial opinions were measured prior to the first Go match on the 9^th^ of March 2016 and was positive over Sedol Lee’s winning against AlphaGo. The low-prior group includes those whose prior beliefs were negative. The following two patterns, which was normally expected, may merit discussion.

First, the prediction about the outcome of the first game apparently followed each person’s prior belief over the Go-capability of Sedol Lee. Those in the high-prior group tended to predict Lee’s winning against AlphaGo, whereas those in the low-prior group tended to predict the opposite. Second, once the outcome of the first game was observed, the participants reacted to the publicly available information and updated their prediction in a similar manner. Most of them leaned towards AlphaGo.

**Fig 3:**
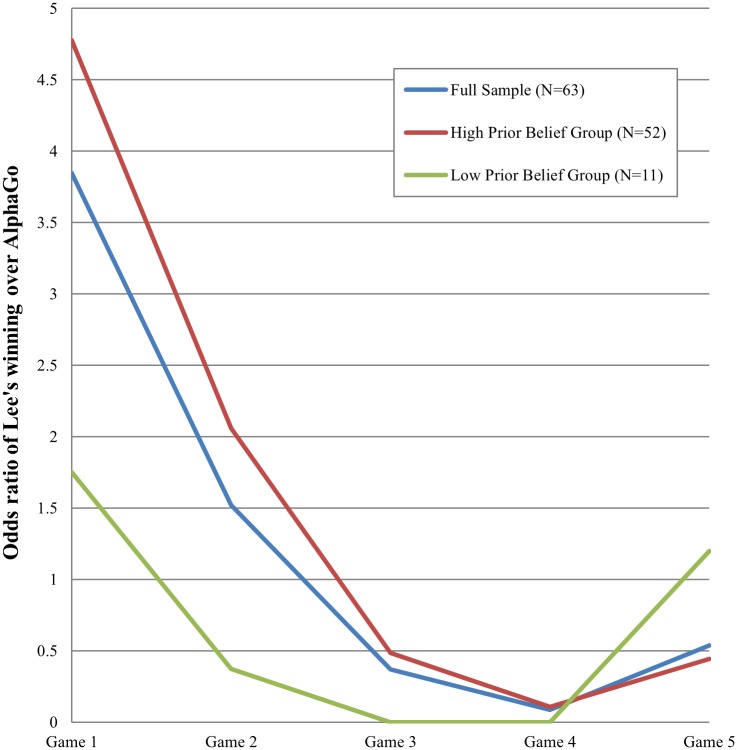
Temporal variation in the prediction of Lee’s winning. Y axis refers to the odds ratio of those who predicted Lee’s winning over AlphaGo. The number of participants (*N*) is 63.

**Fig 4:**
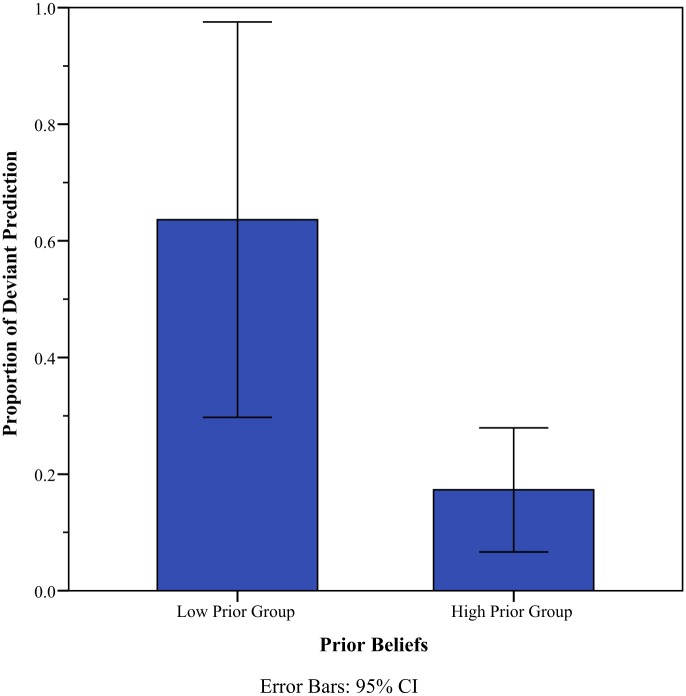
Deviation from initial opinions in the prediction of the first game. The response variable is the proportion of the participants whose prediction for the first game was opposite their prior beliefs. *N* = 63.

Fig 3 summarizes the above-mentioned pattern. It depicts a temporal variation in the odds ratio of the prediction that Lee would win a game. It captures the relative proportion of the participants (*N* = 63) who predicted Lee’s winning against AlphaGo in a given game. For the first game, individuals’ predictions were mostly consistent with their prior beliefs. Interestingly, the odds ratio for the low-prior group was above one, indicating that despite their prior beliefs, those in the low-prior group mostly deviated from initial opinions and predicted Lee’s winning in the first game. The Pearson’s chi-squared test for the proportion that individuals deviated from their prior beliefs in the prediction of the first game is 10.29(*p* = 0.0013). It indicates that those in the high-prior group tended to stick to their initial opinions whereas those in the low-prior did not in the prediction of the first game (Fig 4).

### Variation in outcome prediction and social networks

We then proceeded to examine the role of social relations in the course of predicting the outcome of the Go match between AlphaGo and Sedol Lee. A total of 46 participants were engaged in the prediction of the Go-match in March and responded to the survey of social relations in January. Three of them failed to report their indirect ties so that the values of their ego network density were missing. The final sample for AlphaGo match prediction was 43. We first split the participants into two groups according to ego network density, which reflected the structure of social relations that involved the focal person. The high-density group (*N* = 17) included those whose ego network density was larger than or as high as 0.5. The remaining was included in the low-density group (*N* = 26). Note that relative to the sample of the Go-match prediction, 20 participants were missing in this sample. The proportion of females in these 20 missing observations was similar to that in the final sample (*t* = 0.21, *p* = 0.84). They were mostly high prior-belief holders (the proportion of the high priors was 0.95). Nonetheless, this may not affect our analysis because the proportion of high prior-belief holders was comparable between the two subsamples as follows.

The two groups were highly comparable along such dimensions as prior beliefs in Lee’s capability, gender and the degree of media exposure during the match (Table 1). Those in the high-prior group dominated in both density groups. The proportion of the high-prior group members was 0.765 for the high-density group and 0.769 for the low-density one. The difference was statistically negligible (*t* = 0.03, *p* = 0.97). Similarly, the proportion of females did not differ substantially. It was 0.35 for the high-density group and 0.38 for the low-density group. The degree of media exposure during the match was also similar as well. Both groups watched approximately one out of the five games (1.18 for the high-density group and 1.38 for the low-density one) and read news about the outcomes of the three games (3.65 for the high-density one and 3.50 for the low-density one).

**Table 1:** Demographic characteristics of the study sample.

Variable	Low Density	High Density	*t* statistic
	(*N* = 26)	(*N* = 17)	
	Mean (s.d.)	Mean (s.d.)	
Proportion of high prior belief holders	0.77(0.43)	0.77(0.44)	0.03
Proportion of female participants	0.38(0.49)	0.35(0.49)	0.21
Media exposure			
TV	1.38(1.63)	1.18(1.42)	0.44
Other media	3.50(1.10)	3.65(1.27)	-0.39
Knowledge of AI*	5.21(1.76)	4.74(2.07)	0.76
Knowledge of Go game*	3.42(2.04)	2.90(1.97)	0.84
Emotional response to the 1^st^ game*	6.38(2.19)	6.94(1.95)	-0.87
Degree Centrality	6.19(2.41)	4.82(2.09)	1.97**

Numbers are rounded off at the third decimal place

* Questionnaires available in S2 Text

** *p* = 0.056

**Fig 5:**
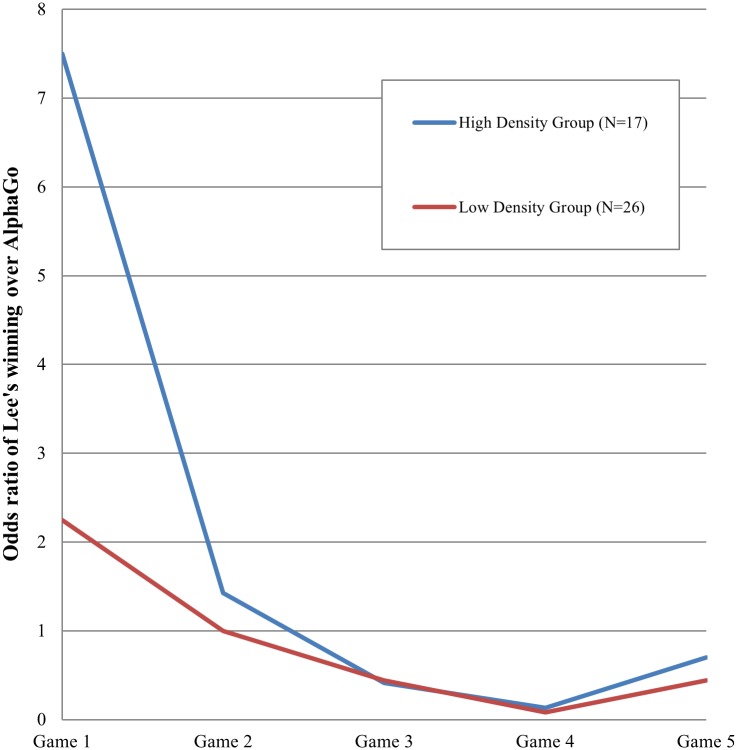
Network density and the prediction of Lee’s winning. Y axis refers to the odds ratio of the proportion of those who predicted Lee’s winning. *N* = 43.

**Fig 6:**
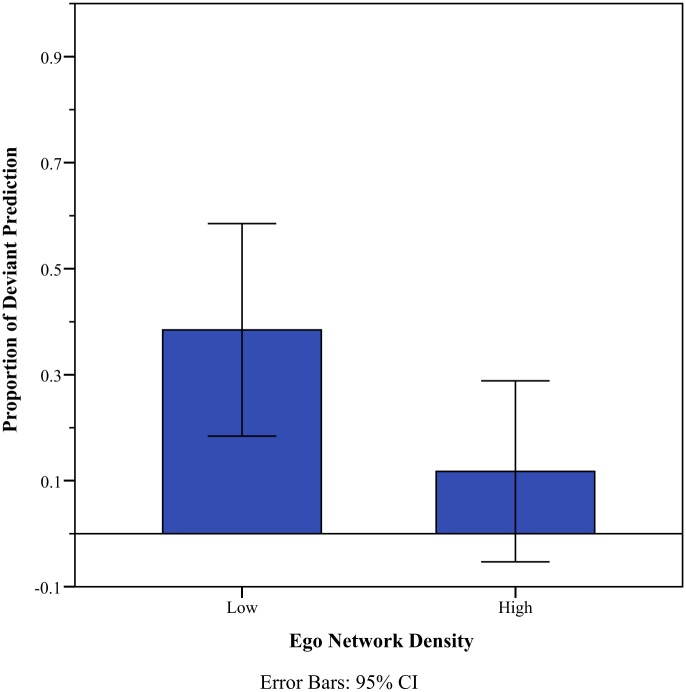
Network density and deviation to initial opinions. The response variable is the proportion of the participants whose prediction for the first game was opposite their prior beliefs. *N* = 43.

Despite apparent similarity between the two density groups, especially with respect to the prior beliefs in Lee’s capability, the prediction of each game differed between the groups. In particular, the prediction of the high-density group resembled that of the high-prior one, whereas the prediction of the low-density group resembled that of the low-prior one (Fig 5). For the prediction of the first game, for example, those whose prediction deviated from their prior beliefs were an 11.8 percent for the high-density group and a 38.5 percent for the low-density one (*χ*^2^(1) = 3.64, *p* = 0.0056, Fig 6). In other words, the high-density group tended to stick to their initial opinions, a pattern that was found in the high-prior group of Fig 4. In comparison, the low-density group tended to update their prediction adaptively in response to information available prior to the match in March (S2 Text). This tendency to adaptively update one’s outcome prediction was apparently associated with the quality of prediction, i.e., accuracy. The low-density group appeared to enjoy a vision advantage for uncertain events compared with the high-density group. The proportion of persons correctly predicting the outcome of the first game was 0.31 for the low-density group and 0.12 for the high-density group.

**Fig 7:**
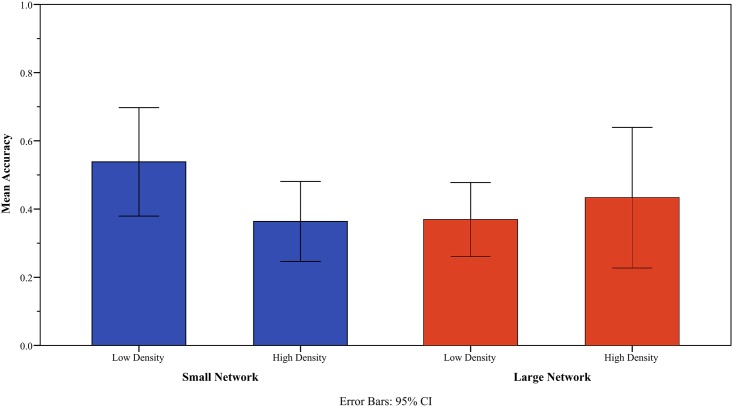
Network density, network size and prediction accuracy. The response variable is the average rate of predicting correctly the outcomes of the Go match. *N* = 43.

Note that there was little group-difference in the rate of correctly predicting the outcome of the whole match (0.45 for the high-density group and 0.39 for the low-density group). This implies that the two groups may not differ in the way of utilizing new information that is publicly available, i.e., a public signal, such as the outcomes of the previous games. One notable pattern however was uncovered when we evaluated the behavior of the two density groups against network size, i.e., the number of task advice friends (Fig 7). For the rate of correct prediction, the low-density group appeared to outperform the high-density one when the network size of the two density groups was smaller than the sample mean of network size, i.e., 5.65 (*t* = 1.94, *p* = 0.065). Yet, such group difference became negligible when the network size of the two density groups was larger than the same mean (*t* = -0.68, *p* = 0.51). To the extent that the low-density group enjoyed a vision advantage when their relations were small-sized, this implies that the disadvantage of the high-density group would be mitigated by having large-sized social relations.

### Decision style variation

**Fig 8:**
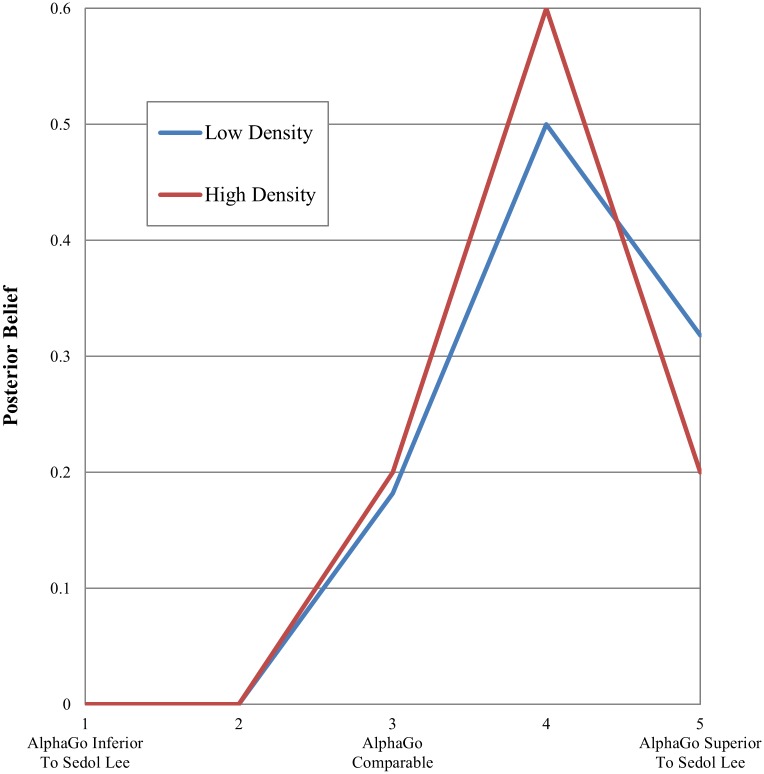
Posterior belief over the go capability of Sedol Lee. Y axis refers to a proportion of the participants for a given level of posterior belief in Lee’s capability. *N* = 37.

In one month after the highly surprising Go match, when the social attention in Korea was given to heated debate over the capability of artificial intelligence in a computerized economy, we re-took a measure of each participant’s belief over the Lee’s capability vis-à-vis AlphaGo, i.e., posterior belief (Fig 8). Six observations were missing in April and yet they did not differ significantly from the sample of the remaining 37 observations with respect to the proportion of high-prior individuals and ego network density. The *t*-test statistics for the difference in high-priors and network density were 0.24 (*p* = 0.82) and -1.28 (*p* = 0.23), respectively. In other words, the non-response bias apparently was not severe in this sample. Given small sample size, caution needs to be taken for statistical inference. The motivation of this analysis here was therefore to evaluate a possible source of the observed variation in social inference against well-known regularities in decision making.

As expected, participants in April (*N* = 37) exhibited a change in their opinions over Lee’s capability. They updated their beliefs in response to a ‘public’ signal, which was the observed result of the match in March. Indeed, none of them sustained their prior beliefs in Sedol Lee and approximately an 80 percent of them reported that AlphaGo was slightly or highly superior to Sedol Lee. A twenty percent of the high-density group as well as a thirty two percent of the low-density group reported that AlphaGo was highly superior to Sedol Lee. This indicates that the high-density group was relatively slow at updating their beliefs. Nonetheless, the both groups found the result of the match to be unexpected and surprising. The average value of a nine-point Likert scale for the perceived surprise of the match outcome was 6.94 for the high density group and 6.38 for the low density one. This means that the both groups perceived the result of the match, i.e., a public signal, in a similar manner. This in turn suggests that the adaptive inference of the low-density group in Fig 6 should have something to do with their way of assessing and interpreting ‘private’ signals whose accessibility varied across individuals and which served as a clue to the outcome of the match in March.

For a better understanding of each participant’s way to assess and make inferences about social events, we undertook the following analyses. First, we employed measures of IPSAQ that has 32 items [57] and examined the difference in attribution style between the two density groups (Table 2). For each item, the participant was asked to judge whether a given event is caused by “something about you, something about the other person, or something about the situation”. Items of the negative events include the following: “A friend refused to talk to you”, “A friend thinks you are unfriendly”, and “A friend ignored you”.

**Table 2:** Attribution style and network density.

	Group				*t*	*p* -value
	Low density		High density			
	(*N* = 22)		(*N* = 15)			
	*Mean*	s.d	*Mean*	s.d.		
**Positive items**						
•Internal	11.41	3.54	9.93	4.91	1.06	0.29
•Personal	1.09	1.63	3.33	3.6	-2.26*	0.04
•Situational	3.5	3.19	2.73	2.4	0.79	0.44
**Negative items**						
•Internal	7.95	2.89	9.67	4.01	-1.51	0.14
•Personal	2.09	2.16	2.8	3.03	-0.83	0.41
•Situational	5.95	3	3.53	2.97	2.42*	0.02
**Externalizing Bias**	3.45	4.54	0.27	5.35	1.95†	0.06
**Personalizing Bias** ^a^	0.25	0.24	0.41	0.31	-1.69	0.1
**Whole items**						
•Internal	19.36	4.59	19.6	7.19	-0.12	0.9
•Personal	3.18	3.63	6.13	5.97	-1.87	0.1
•Situational	9.45	4.19	6.27	4.08	2.29*	0.03

^†^ *p* < .1;

* *p* < .05

^a^ The denominator for three observations were zero and thus were not included in the computation of personalizing bias.

For the negative events, the low-density group was more likely than the high-density group to make situational attributions (*t* = 2.42, *p* = 0.02). In other words, they apparently did not blame themselves for negative events. Moreover, the low-density group exhibited a strong tendency for externalizing bias when compared to the high-density group (*t* = 1.95, *p* = 0.06). Note that externalizing bias is defined as follows: the number of internal attributions for positive events minus the number of internal attributions for negative events. Accordingly, a positive value of this bias indicates that a person tends to blame oneself less for negative events. This person avoids negative self-attributions. Externalizing bias is therefore associated with self-serving bias. Note that individuals tend to make situational attributions for negative events when they are embedded in individualistic cultures rather than in collectivistic ones [59]. In comparison, those in a collectivistic culture would be less likely to make internal attribution for positive events. For example, they may accept positive events as their fate or destiny. If this is the case, our data may suggest that the behavior of the low-density group would be comparable to that of individualistic cultures.

**Fig 9:**
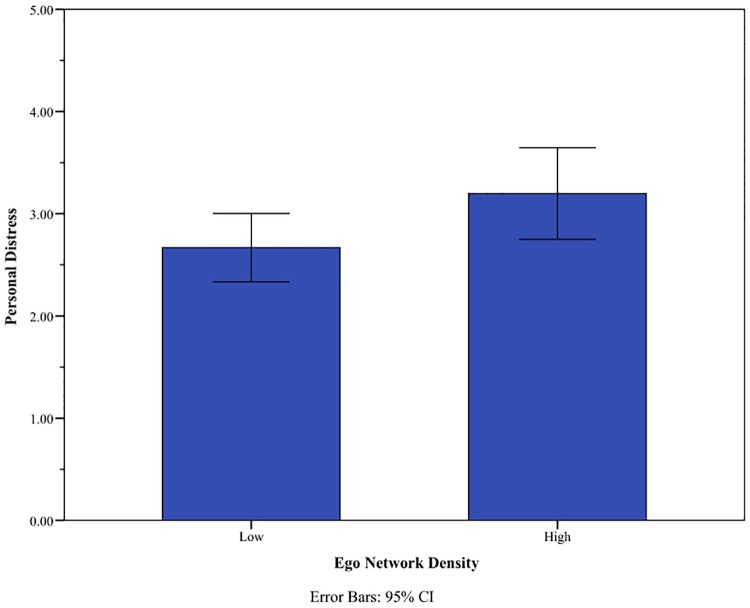
Personal distress and network density. The mean response of the Personal Distress scale of IRI is reported. *N* = 35 (the number of observations for low density is 22 whereas that for high density is 13).

Along this line, the analysis of self-oriented feelings in interpersonal settings made a similar implication. Owing to missing values, a total of 35 observations were used for the analysis. Among four subscales of the IRI measure, the scale of personal distress alone yielded a noticeable difference between groups. The *t*-test statistics for Perspective Taking, Emotional Concern, and Fantasy scales were -0.37, -0.51 and 0.72, respectively. On the measure of personal distress, the low-density group reported a lower score than the high-density group (*t* = -2.03, *p* = 0.053), implying that the low-density group would be less sensitive to emotionally challenging situations of the others (Fig 9). In other words, the low-density group appeared to stand aloof even though they maintained durable relationships with others.

## Replication tests

The analysis of the AlphaGo match case indicated a role of a person’s ego network in the inference of an uncertain social event. However, the unique nature of the Go match as well as our sample size may limit the external validity of our finding. To mitigate these concerns, we ran replication tests that involved prediction tasks across different social situations. With a new approval from IRB at Seoul National University (IRB no. 1604/003-014), we were allowed to recruit a new pool of participants for multiple experiments with a contract starting in May 2016. From a total of 136 undergraduate students that were recruited, 95 participants responded to our social network survey that was administered from October 31, 2016 to November 18, 2016. Five of them were also involved in the AlphaGo study. For those who participated in the network survey, we ran three sets of replication tests. A total of 84 persons agreed to participate in our tests. Ego network density was not defined for 5 participants whose ego network size was one. Accordingly, the final sample for replication tests included the observations of 79 participants (34 females and 45 males; aged between 18 and 24) (S2 Text).

### Prediction tasks

On the 25^th^ of November 2016, we designed an online questionnaire and asked the participants to predict the outcome of uncertain social events: the winners of the Blue Dragon Award (a local film festival), the vote of the Korean parliament to impeach President Park and the counterfactual assessment of Bernie Sanders to win the US presidential election. These tests were designed to evaluate individual difference in predicting tasks for two domains, artworks and politics. It took no more than 20 minutes to answer the tests.

For the film awards, we asked the participants to predict the 2016 winners of best picture, directing and actor and actress in a supporting role. We collected their predictions prior to the opening of the film festival at 8:00 pm on the 25^th^ of November. We expected that consensus would develop as to the winners of actor and actress in a leading role, who were more known to the public than actors and actresses in a supporting role. We thus did not test the prediction of the winners of actor and actress in a leading role.

For domestic politics in Korea, we asked whether the Korean parliament voted to impeach President Park by December 9, 2016. In November, hundreds of thousands of citizens marched on every Saturday against President Geun-hye Park, who was allegedly responsible for a series of political corruptions that the prosecutors in Korea were examining. Indeed, on November 25, a Gallup Korea poll showed that President Park’s approval rating was a 4%, which indicated her political status among citizens. On the other hand, the president herself denied wrongdoing and opposition parties had yet to persuade the reluctant ruling party to vote to impeach the president. Accordingly, it was highly uncertain whether the parliament voted to impeach until the 9^th^ of December when the session of the parliament in year 2016 was scheduled to end.

For the 2016 US presidential election where Hillary Clinton lost the election to Donald Trump, we asked whether Bernie Sanders would have won the 2016 presidential election if he had been the candidate for the Democratic Party. It was designed to uncover the variation in decision-making styles between the high and low-density groups. We drew on the availability bias, which has been extensively tested [60]. Participants were randomly assigned to either of the two prediction scenarios. Those in the Sanders scenario were primed with an article that described Bernie Sanders’s speech after the 2016 presidential election, whereas those in the Clinton scenario were primed with an article that reported Hillary Clinton’s speech after the presidential election. Insofar as the availability of congruent information helps increase the subjective probability of an event [61,62], the predicted probability of Sanders to win the election would be higher for those in the Sanders scenario than in the Clinton scenario. Given the availability bias, we examined whether ego network density would aggravate or weaken the bias.

Like the AlphaGo match case, these prediction tasks have the following features. First, there was no correct answer available to the participants as well as experimenters at the time of prediction. Second, participants were asked to predict an unknown state of a social event. Their prediction was a form of extrapolation by estimating an event outside its current state that was partially known to the participants. To this end, they extrapolated their prior knowledge of the event to a novel situation. Given their knowledge of the Go capability of Sedol Lee, the popularity of films, domestic and overseas politics, they sought to understand the competition with an unknown machine, i.e., AlphaGo, the decisions of experts such as film critics and politicians, and a counterfactual assessment of the US election.

### Social networks

Online and offline social relations of participants were measured from October 31, 2016 to November 18, 2016. It took approximately 30 minutes to collect data on social relations from each participant. Prior studies suggest that the offline social relations of college students and adolescents may remain stable at least for short periods, e.g., 6 months [63,64,65]. Accordingly, the following name generator for task-based relations was administered: “for the last one year to whom you go for help and advice on important matters?” For online social relations, participants were asked to carry their smartphones to the experimental room and to report their use of social networking services such as Facebook while referring to their smartphones.

The name generator for offline relations asked participants to name up to five persons with whom they discussed important matters. This instrument of naming only five names helps uncovering significant others in a person’s immediate social relations in a way that alleviates the cognitive burden of recalling all the names of acquaintances [55,56]. The rationale for five-person naming is that the citation of more than five names may not increase the chance to recall significant others who are non-redundant contacts [56]. This instrument by design tends to under-estimate the density of a person’s ego network.

For the sample of 79 participants, the mean of ego network density was 0.33 (s.d. = 0.27), which was lower relative to the sample of the AlphaGo study (Table 3). For a better understanding of the replication test sample, we drew on the sample mean and split the observations into three subgroups: low, middle and high density groups. The following three patterns merit attention. First, there was a high overlap in social contacts between online and offline social relations, irrespective of ego network density. For those who were Facebook users (*N* = 67), the overlap in online and offline social contacts ranged from a 0.53 to 0.69 percent. Indeed a growing body of empirical studies suggests that online social relations overlap with offline ones [7,66,67]. Second, the level of political participation, measured by voting in the general election in April 2016, was relatively high across three groups. In comparison, the high-density group appeared to be an active consumer of films relative to the other groups. Lastly, online social relations of the middle density group were rather distinct from those of the low and high density groups. For example, the use of KakaoTalk, a Korean equivalent of WhatsApp, illustrated this tendency. The users of KakaoTalk normally invite different persons to different group-chatrooms. The number of group-chatrooms that a person opens would then be associated with the number of online social groups that the person maintains. In this regard, the middle density group tends to have larger online social groups for 10-day observation while exhibiting a lower overlap in online and offline social relations. Given this tendency, in the analysis below, we further examined whether the middle-density group would be a source of additional variation in the findings.

**Table 3:** Characteristics of the replication test sample.

Variable	Ego Network Density (*θ*)		
	Low	Middle	High
	(*θ <* 1/3)	(1/3 ≤ *θ* < 1/2)	(*θ* ≥ 1/2)
	Mean (s.d.)	Mean (s.d.)	Mean (s.d.)
*Full sample*	(*N* = 47)	(*N* = 23)	(*N* = 19)
KakaoTalk group-chatrooms last 10 days (number)	14.70(8.42)	15.48(10.23)	13.63(6.36)
*For Facebook users only*	(*N* = 35)	(*N* = 15)	(*N* = 17)
Online & offline contact overlap (proportion)	0.69(0.29)	0.53(0.24)	0.66(0.38)
*For those who participated in replication tests*	(*N* = 44)	(*N* = 17)	(*N* = 18)
Voted in the last general election (1 if yes)	0.61(0.49)	0.59(0.51)	0.67(0.49)
# of watching films at the cinema (per year)	8.25(6.41)	6.06(4.45)	10.28(6.92)
# of watching films on the phone (per month)	3.72(4.66)	3.71(4.81)	5.61(7.71)

Numbers are rounded off at the third decimal place

### Test of artworks

For the prediction of film awards winners, participants apparently were not confident in their predictions, indicating the subjective difficulty of the prediction task (S2 Text). The proportion of correct predictions varied widely depending on the categories of awards (Table 4). Given that the finalists for each award were five, prediction difficulty should be viewed as being high when the success rate of the prediction becomes closer to 0.25, i.e., the effect of pure chance. The average proportion of correct predictions suggested that the prediction of directing award was relatively easy compared to that of actor award (*M*_*directing*_ = 0.44, *M*_*actor*_ = 0.27). This indicates that the reputations of actors and actresses were not useful for the participants to estimate the decisions of critics on the award committee. Alternatively, it is possible that there would be no consensus as to the reputations of actors and actresses in a supporting role. In comparison, a higher proportion of correct prediction for directing suggests that the views of the participants apparently coincided with those of critics regarding the award of directing.

**Table 4:** Social networks and the correct prediction of film award winners.

Award Category	Sample Average	Low Density	Middle + High Density
	(*N* = 79)	(*N* = 44)	(*N* = 35)
	Mean (s.d.)	Mean (s.d.)	Mean (s.d.)
Directing	0.44 (0.49)	0.43 (0.50)	0.46 (0.51)
Best Picture *	0.33 (0.47)	0.41 (0.49)	0.23 (0.43)
Actress in a supporting role	0.30 (0.46)	0.29 (0.46)	0.31 (0.47)
Actor in a supporting role	0.27 (0.44)	0.29 (0.46)	0.23 (0.43)

Numbers are rounded off at the third decimal place

* *χ*^2^(1) = 2.877, *p* = 0.090 for the difference in proportion between the two groups.

For the prediction of person-related awards such as directing and actor in a supporting role, a person’s ego network density was not associated with the success rate of predicting awards winners. However, the low density group clearly outperformed the other group as to the prediction of best picture award (*χ*^2^(1) = 2.877, *p* = 0.090). Given that the middle-high density group was active consumers of films (Table 3), this was rather unexpected. The most frequent predictions (37.14%) by the middle-high density group was the film with the least sales share in the year, which indicates that this group based their prediction solely on one indicator, i.e., whether the film was commercially successful or not. In comparison, the low density group did not exhibit such behavior. Only 11.36% of the low density group predicted that the film with the least sales share would be the winner (S2 Text).

Although the average size of ego network in the sample was 4, there were seven observations whose network size was two. The average number of KakaoTalk group-chatrooms was 12.3 for these respondents, who frequently went to cinema (*M* = 11.14). It was possible that the observed effects were driven by outliers in network size. Yet, the effects remained consistent even when the seven observations were dropped from the sample (S2 Text).

### Test of politics

The task of the impeachment prediction has the following set-up. The public’s opinion of President Park in November was highly negative, indicated by her approval rate as low as 4%. Yet the parliament’s vote to impeach would be virtually infeasible without the help of the ruling party whose majority still leaned towards the president. Against this background, the participants were asked to predict whether the parliament would vote to impeach the president by December 9, 2016, the last day of the 2016 session.

**Fig 10:**
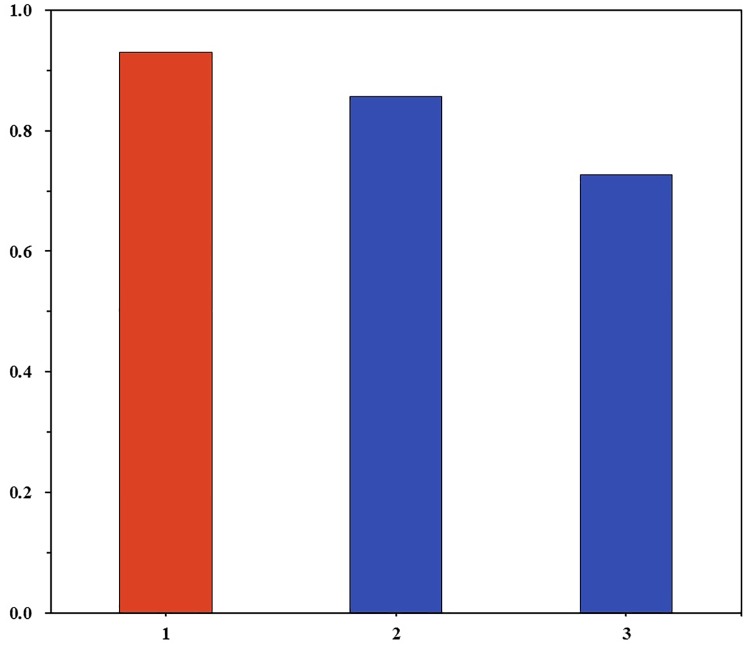
Prospect for impeachment and social networks. 1. Support for impeachment (Gallup Korea Poll on December 9, 2016; aged from 19 to 29) 2. High prospects for impeachment (Middle+High density group) (*N* = 35) 3. High prospects for impeachment (Low density group) (*N* = 44)

The prediction of each participant was measured by a 6-point Likert scale with point 6 being ‘most likely’ and point 1 being ‘least likely’. We coded high prospects for impeachment as one when the participant’s prediction was above or as high as point 4. The chi-squared test did not uncover the difference in proportions between the low and the middle-high density groups at a 0.05 significance level (*χ*^2^(1) = 1.947, *p* = 0.163). Inspection of the data however suggests that the predictions made by each density group apparently did not converge. 72.7% of the low density group had high prospects of the impeachment, whereas the proportions of high prospects were 88.2% for the middle density group and 83.3% for the high density group. Indeed the middle and high density groups were closer to the public’s negative opinion of the president at the time of prediction. For example, a Gallup Korea poll released on December 9, 2016 showed that 93% of the respondents in the 20s who were surveyed from December 6 to December 8 were in support of the impeachment motion (Fig 10). In comparison, the prediction of the low density group deviated from the majority view. This pattern was also found in a logit model of high prospects for the impeachment:

$$\text{log}\left[\frac{p\left(high\;prospects=1\right)}{1-p}\right]=a_{0}\text{+}a_{1}*\theta \text{+}a_{2}*\theta^{2}+\epsilon$$

where *θ* is a person’s ego network density and *ϵ* is the disturbance in the model. The maximum likelihood estimates for *a*_1_ and *a*_2_ were 5.340 (*χ*^2^(1) = 2.864, *p* = 0.091) and –5.348 (*χ*^2^(1) = 3.149, *p* = 0.076), respectively. Note that the likelihood ratio test statistic for the model was rather insignificant. The chi-squared statistic was 3.144 (*p* = 0.208) for the null model of zero coefficients. Although caution needs to be taken for further inference, our data still suggest that the view of the low density group may not represent the majority view in the community.

Lastly, we evaluated possible variations in decision-making styles from the counterfactual predictions for the US presidential election. Each participant’s estimated probability (%) of Bernie Sanders to win the election was slightly higher in the Sanders scenario (*N* = 37, *M*_*Sanders*_ = 48.22) than in the Clinton scenario (*N* = 42, *M*_*Clinton*_ = 44.82). When two observations of zero-probability estimate were dropped, we ran a one-sided *t* test for the difference in the logged probability, which uncovered a mild difference between Clinton and Sanders scenarios (*N* = 77, *t* = -1.6, *p* = 0.059). For the high density group, the average difference in the probability was substantial and -13.17 (*N* = 11, *M*_*Sanders*_ = 52.45 vs *N* = 7, *M*_*Clinton*_ = 39.29). For the middle density group it was 3.63 (*N* = 7, *M*_*Sanders*_ = 47.00 vs *N* = 8, *M*_*Clinton*_ = 50.63), whereas for the low density group it was -2.62 (*N* = 18, *M*_*Sanders*_ = 48.78 vs *N* = 26, *M*_*Clinton*_ = 46.15). Indeed, the one-sided *t* test statistic was -1.67 (*p* = 0.053) when the observations of the middle density were dropped from the sample. This clearly indicates that compared with other density groups, the availability bias was salient among the high density group.

## Discussion

This study examines whether the way that a person makes inferences about unknown events is associated with his or her social relations, more precisely, those characterized by ego network density that reflects the structure of a person’s social relation. From the analysis of individual predictions on the Go match between AlphaGo and Sedol Lee in March 2016, this study shows the following: (1) the high-density group, i.e., those lacking in brokerage opportunities in their social relations, remained loyal to their initial opinions about the Go-capability of Sedol Lee; (2) the high-density group was less likely than the low-density one, i.e., those full of brokerage opportunities in their relations, to correctly predict the outcome of the first game; and (3) the high-density group tended to be sensitive to others’ distress, measured by the Personal Distress scale, whereas the low-density group tended to exhibit the externalizing bias in attribution styles, measured by the Internal, Personal and Situational Attributions Questionnaire. This finding suggests that network density is negatively associated with vision advantage.

Given that this Go match gained a much attention from the society and that the majority prior to the match was in favor of Sedol Lee, the observed behavior of the low-density group suggests that relative to the high-density group, the low-density one would likely incorporate the minority and thus dis-conforming view into their inferences, making balanced predictions. Indeed, the analysis of attribution styles as well as self-oriented feelings suggests that the low-density group may behave as if they were stand-alone and relational-free. Individuals in the low-density group are well embedded into their social relations and yet enjoy autonomy from such relations, as Burt already noted in his analysis of structural holes in social relations [3].

The prediction of social events in the near future is interactive in nature such that individuals are directly or indirectly involved in the evolution of social events. For example, those who predict popular artworks or election winners need to distinguish between who they prefer and who the others prefer. They even need to consider the possibility that their preference may influence the opinions of others that are connected to them. To the extent that experts take into account the views of non-experts, the same consideration may apply to the prediction of experts’ decisions such as film awards. In this regard, the prediction of the AlphaGo’s match has much in common with that of other social events for the following reasons.

First of all, it is basically a task of predicting a person’s capability, a situation that is commonly found in hiring decisions at the workplace. However, a unique feature of the AlphaGo case is that participants were asked to predict the performance of a player in a novel situation, i.e., a match with a non-human opponent whose capability was not fully disclosed. Accordingly, the past track records of Sedol Lee were neither relevant to nor useful for the prediction of the match outcome, which was highly uncertain. Moreover, it was likely that a society-level interest in the match would either motivate the player or add a burden on the player. In short, it was an uncertain social event.

Along this line, our study suggests that a person with low ego-network density is more likely to enjoy a vision advantage in the prediction of uncertain social events than his or her counterpart with high density. Although individuals with a low density network are well embedded in social relations, they may arguably avoid following the herd, i.e., a majority view. When our finding at the individual level is extended to the studies on prediction market [14,15], it implies that ego network density may serve to mitigate or aggravate the social ‘influence’ by others. Given that our study examines social ‘inference’, a boundary condition for this study hinges upon the observation that a person’s actual behavior or choice does not necessarily reflect his or her own belief [68,69]. Accordingly, our study does not suggest that a low-density person is free from social influence. It does not exclude a possibility that a low-density person imitates the choice of the majority while believing otherwise at heart. Rather, it only suggests that the inference of a low-density person as to uncertain social events would be insensitive to the view of the majority.

When it comes to opinion formation in a social network, attention has been given to a process in which apparently dis-organized choices at the individual level aggregate into organized collective ones [44,45,47,70,71]. While individuals respond to the choices of others that are limitedly observable, a society-wide consensus or trend emerges in a way that is not easily anticipated by each individual choice [71,72]. The structure of global network that dictates whom to interact with is one of factors that govern the process of aggregating individual opinions and choices into global regularities. Although we limit analysis to a local structure of global network, namely, a person’s ego network, our study complements this line of research tradition as follows.

First, the type of a person’s ego network, measured by ego network density, may indicate his or her location in a global network of multiple, partially-overlapping groups. Real-life social relations are locally clustered such that the interactions of individuals are not at random and that multiple groups of frequently-interacting individuals, i.e., communities, may constitute the global network of a collectivity in question [33,35,48,73,74]. Indeed, sociologists address structural differentiation at the society level and opt for a status-based model of social exchange that corresponds to the community structure of complex networks [75,76]. To the extent that non-redundant contacts are drawn from different social groups, a person with a low-density ego network, i.e., a network broker, is likely to have multiple memberships in social networks. The model of strength of weak ties [10] as well as the network brokerage model [3] presumes that a network broker connects otherwise disconnected groups. The locational advantage of a network broker translates into social influence that a broker exerts over his or her social contacts [11,12]. In comparison, a high-density person, i.e., a non-broker, is likely to be located at the center of a locally cohesive group or a community. Lacking in brokerage opportunities, this person would be rather disconnected from the rest of the global network. Of course, the density of a person’s ego network could be a noisy proxy for the locational advantage of a network broker [77]. A broker at the core of a global network may weigh more than his or her counterpart in the periphery of the network [78]. Accordingly, the structure of global network may amplify or suppress the locational advantage of a network broker [36,74].

Second, our analysis of ego network however deviates from opinion formation in complex networks. We distinguish between social influence by others and social inference by the individual him- or herself. The latter is the focus of our analysis. Compared with the analysis of consensus-building at the global network level, our focus is on individual ideation at the ego network level, i.e., how individual opinions develop given the influence of his or her immediate social relations. To the extent that network brokers are the drivers of opinions formation at the global level [10,36], the view of non-network brokers should resemble those of the brokers. However, our findings suggest otherwise. The views of the brokers tend to diverge from those of non-brokers. They often deviate from the herd as was the case with the AlphaGo and other three tests of this study. This at least implies that opinion formation at the global level may draw on two distinct sources at the local level, i.e., network brokers and non-brokers, whose contributions to opinion formation may not be identical [22,36].

This study is not without limitation. From a focal person’s perspective, his or her immediate social relations may become significant others only when they are readily available in his or her memory. However, unspecified measurement errors associated with self-generating reports such as name generators may limit the inference about the role of ego networks. Regarding this, a recent study of wearable sensor data merits attention, which strongly indicates that long-term relationships are likely to be captured in the self-generating reports [66]. Given the on-going social relations, we examined the structure of ego networks that help improve decision making under uncertainty. Although we found the variation in attribution styles and availability heuristic between high and low density groups, our focus was mainly on the outcome of decisions, not the process, which demands additional tests of cognitive tasks. Another issue is about online social networks. Given a relatively high overlap in significant others between offline and online social relations in our sample, the effects found in offline relations would be applicable to the working of online relations. Nonetheless, offline social relations differ substantially from online ones in that face-to-face interactions underlie offline social relations. Owing to the overlap between offline and online social networks, it is rather difficult to separate the observed effects of offline relations from those of online relations. This separation requires a different experimental design, which is beyond the current scope of this study.

Research on the role of social networks in social inference has been mainly focused on the limited size of social networks that arises from a person’s cognitive constraints [7,27,79]. In this study, we take one step further and suggest that not only the size of a person’s social network but also the structure of a network, i.e., ego network density, may underlie the individual variation in social inference. Future research awaits further refinements in the working of ego network density across various domains of social inference.

## Supporting information

S1 Dataset This file contains the raw data used in this study. (XLSX)

S1 Text This file gives the definition of each variable used in this study. (DOCX)

S2 Text This file illustrates the composition the participants, the details of the figures, a set of questionnaires used in this study and the variation in the public exposure of the internet users in Korea to the Go-match between AlphaGo and Sedol Lees in 2016. (DOCX)

## References

[1] Fiedler K. 2000 Beware of samples! A cognitive-ecological sampling theory of judgment biases. Psychol Rev, 107: 659–676. 11089402 10.1037/0033-295x.107.4.659

[2] Gigerenzer G., Hoffrage U., & Kleinbolting H. 1991 Probabalistic mental models: A Brunswikian theory of confidence. Psychol Rev, 98: 506–528. 1961771 10.1037/0033-295x.98.4.506

[3] Burt R.S. 1992 Structural Holes. Cambridge, MA: Harvard University Press.

[4] Jackson M.O. 2008 Social and Economic Networks. Princeton, NJ: Princeton University Press.

[5] Watts D.J. 2004 The “new” science of networks. Annu Rev Sociol, 30: 243–270.

[6] Podolny J.M. 2001 Networks as the pipes and prisms of the market. Am J Sociol, 107: 33–60.

[7] Dunbar R.I.M., Arnaboldi V., Conti M., & Passarella A. 2015 The structure of online social networks mirrors those in the offline world. Soc Networks, 43: 39–47.

[8] McPherson M., Smith-Lovin L., & Cook J.M. 2001 Birds of a feather: Homophily in social networks. Annu Rev Sociol, 27: 415–444.

[9] Kossinets G., & Watts D.J. 2009 Origins of homophily in an evolving social network. Am J Sociol, 115: 405–450.

[10] Granovetter M.S. 1973 The strength of weak ties. Am J Sociol, 78: 1360–1380.

[11] Stovel K., & Shaw L. 2011 Brokerage. Annu Rev Sociol, 38: 139–158.

[12] Goyal S., & Vega-Redondo F. 2007 Structural holes in social networks. J Econ Theory, 137: 460–492.

[13] Salganik M.J., Dodds P.S., & Watts D.J. 2006 Experimental study of inequality and unpredictability in an artificial cultural market. Science, 311: 854–856. 10.1126/science.1121066 16469928

[14] Lorenz J., Rauhut H., Schweitzer F., & Helbing D. 2011 How social influence can undermine the wisdom of crowd effect. Proc Natl Acad Sci U S A, 108(22):9020–9025. 10.1073/pnas.1008636108 21576485 PMC3107299

[15] Hong L., & Page S.E. 2004 Groups of diverse problem solvers can outperform gropus of high-quality problem solvers. Proc Natl Acad Sci U S, 101(46):16385–16389. 10.1073/pnas.0403723101 PMC528939 15534225

[16] Aral S., & Van Alstyne M. 2011 The diversity-bandwidth trade-off. Am J Sociol, 117: 90–171.

[17] Bae J., & Koo J. 2008 Information loss, knowledge transfer cost and the value of social relations. Strategic Organization, 6: 227–258.

[18] Coleman J.S. 1988 Social capital in the creation of human capital. Am J Sociol, 94:S95–S120.

[19] Obstfeld D. 2005 Social networks, the tertius lungens orientation, and involvement in innovation. Adm Sci Q, 50: 100–130.

[20] Reagans R., & McEvily B. 2003 Network structure and knowledge transfer: The effects of cohesion and range. Adm Sci Q, 48: 240–267.

[21] Buskens V., Raub W., & Van der Veer J. 2010 Trust in triads: An experimental study. Soc Networks, 32: 301–312.

[22] Centola D. 2010 The spread of behavior in an online social network experiment. Science, 329: 1194–1197. 10.1126/science.1185231 20813952

[23] Carnabuci G., & Dioszegi B. 2015 Social networks, cognitive style, and innovative performance: A contingency perspective. Acad Manage J, 58: 881–905.

[24] Larrick R.P., Mannes A.E., & Soll J.B. 2011 The social psychology of the wisdom of crowds In Krueger J.I. (Ed.), Frontiers in Social Psychology: Social Judgment and Decision Making. New York, NY: Psychology Press.

[25] Sanfey A.G. 2007 Social decision-making: Insights from game theory and neuroscience. Science, 318: 598–602. 10.1126/science.1142996 17962552

[26] DiMaggio P., & Garip F. 2011 Network effects and social inequality. Annu Rev Sociol, 38: 93–118.

[27] Burt R., Kilduff M., & Tasselli S. 2013 Social network analysis: Foundations and frontiers on advantage. Annu Rev Psychol, 64: 527–547. 10.1146/annurev-psych-113011-143828 23282056

[28] Boccaletti S., Latora V., Moreno Y., Chavez M., & Hwang D.-U. 2006 Complex networks: Structure and dynamics. Phys Rep, 424: 175–308.

[29] Newman E.M.J. 2010 Networks: An Introduction. NY: Oxford University Press.

[30] Watts D.J., & Strogatz S.H. 1998 Collective dynamics of ‘small-world’ networks. Nature, 393: 440–442. 9623998 10.1038/30918

[31] Barabasi A.-L., Albert R. 1999 Emergence of scaling in random networks. Science, 286: 509–512. 10521342 10.1126/science.286.5439.509

[32] Amaral L.A.N., Scala A., Barthelemy M., & Stanley H.E. 2000 Classes of small-world networks. Proc Natl Acad Sci U S A, 97(21):11149–11152. 10.1073/pnas.200327197 11005838 PMC17168

[33] Girvan M., & Newman M.E.J. 2002 Community structure in social and biological networks. Proc Natl Acad Sci U S A, 99(12):7821–7826. 10.1073/pnas.122653799 12060727 PMC122977

[34] Guimera R., Sales-Pardo M., & Amaral L.A.N. 2007 Classes of complex networks defined by role-to-role connectivity profiles. Nat Phys, 3: 63–69. 10.1038/nphys489 18618010 PMC2447920

[35] Lancichinetti A., Radicchi F., & Ramasco J.J. 2010 Statistical significance of communities in networks. Phys Rev E, 81: 046110. 10.1103/PhysRevE.81.046110 20481789

[36] Watts D.J. 2002 A simple model of information cascades on random networks. Proc Natl Acad Sci U S A, 99(9):5766–5771. 10.1073/pnas.082090499 16578874 PMC122850

[37] Lopez-Pintado D. 2008 Diffusion in complex social networks. Games Econ Behav, 62: 573–590.

[38] Centola D., & Macy M. 2007 Complex contagions and the weakness of long ties. Am J Sociol, 113(3): 702–734.

[39] Nematzadeh A., Ferrara E., Flammini A., & Ahn Y.-Y. 2014 Optimal network modularity for information diffusion. Phys Rev Lett, 113: 088701 10.1103/PhysRevLett.113.088701 25192129

[40] Burt R. 2010 Neighbor Networks NY: Oxford University Press.

[41] Fang A., Landis B., Zhang Z., Anderson M.H., Shaw J.D., & Kilduff M. 2015 Integrating personality and social networks: A meta-analysis of personality, network position, and work outcomes in organizations. Organ Sci, 26(4): 1243–1260.

[42] Krackhardt D. 1990 Assessing the political landscape: Structure, cognition, and power in organizations. Adm Sci Q, 35(2): 342–369.

[43] Gale D., & Kariv S. 2003 Bayesian learning in social networks. Games Econ Behav, 45: 329–346.

[44] Granovetter M. 1978 Threshold models of collective behavior. Am J Sociol, 83(6): 1420–1443.

[45] Javarone M.A. 2014 Network strategies in election campaigns. J Stat Mech, P08013.

[46] Podolny J.M., & Baron J.N. 1997 Resources and relationships: Social networks and mobility in the workplace. Am Sociol Rev, 62(5): 673–693.

[47] Burt S. 2004 Structural holes and good ideas. Am J Sociol, 110(2): 349–399.

[48] Fleming L., Ming S., & Chen D. 2007 Collaborative brokerage, generative creativity and creative success. Adm Sci Q, 52: 443–475.

[49] Tortoriello M., & Krackhardt D. 2010 Activating cross-boundary knowledge: The role of Simmelian ties in the generation of innovations. Acad Manage J, 53(1): 167–181.

[50] Wolfers J., & Zitzewitz E. 2004 Prediction markets. J Econ Perspect, 18(2): 107–126.

[51] Majumder S.R., Diermeier D., Rietz T.A., & Amaral L.A.N. 2009 Price dynamics in political prediction markets. Proc Natl Acad Sci U S A, 106(3):679–684. 10.1073/pnas.0805037106 19155442 PMC2630077

[52] Campbell K.E., Lee B.A. 1991 Name generator in surveys of personal networks, Soc Networks, 13: 203–221.

[53] Fowler J.H., & Christakis N.A. 2008 Dynamic spread of happiness in a large social network: Longitudinal analysis over 20 years in the Framingham Heart Study. Br Med J, 337(a2338): 1–9. 10.1136/bmj.a2338 PMC2600606 19056788

[54] Feld S.L. 1981 The focused organization of social ties. Am J Sociol, 86: 1015–1035.

[55] Marsden P. 2005 Recent developments in network measurement In Carrington P.J., Scott J., Wasserman S. (eds.). Models and Methods in Social Network Analysis: 8–30. NY: Cambridge University Press.

[56] Merluzzi J., & Burt R. 2013 How many names are enough? Identifying network effects with the least set of listed contacts. Soc Networks, 35: 331–337.

[57] Kinderman P., & Bentall R.P. 1996 A new measure of causal locus: the internal, personal and situational attributions questionnaire. Pers Individ Dif, 20: 261–264.

[58] Davis M.H. 1983 Measuring individual differences in empathy: Evidence for a multidimensional approach. J Pers Soc Psychol, 44: 113–126.

[59] Triandis H.C., & Gelfand M.J. 2012 A theory of individualism and collectivism In Lange P. A. M., Kruglanski A. W., & Higgins E. T. (eds.), Handbook of Theories of Social Psychology, 2: 498–520. London: Sage.

[60] Kahneman D., & Tversky A. 1973 On the psychology of prediction. Psychol Rev, 80(4): 237–251.

[61] Carroll J.S. 1978 The effect of imagining an event on expectations for the event: An interpretation in terms of the availability heuristic. J Exp Soc Psychol, 14(1): 88–96.

[62] McKelvie S.J. 1997 The availability heuristic: Effects of fame and gender on the estimated frequency of male and female names. J Soc Psychol, 137(1): 63–78. 10.1080/00224549709595414 9121143

[63] Nezlek J.B. 1993 The stability of social interaction. J Pers Soc Psychol, 65(5): 930–941.

[64] Berndt T.J., & Hoyle S.G. 1985 Stability and change in childhood and adolescent friendships. Dev Psychol, 21(6): 1007–1015.

[65] Neckerman H.J. 1996 The stability of social groups in childhood and adolescence: The role of the classroom social environment. Soc Dev, 5(2): 131–145.

[66] Mastrandrea R., Fournet J., & Barrat A. 2015 Contact patterns in a high school: A comparison between data collected using wearable sensors, contact diaries and friendship surveys. PLoS One, 10(9): e0136497 10.1371/journal.pone.0136497 26325289 PMC4556655

[67] Reich S.M., Subrahmanyam K., & Espinoza G. 2012 Friending, IMing, and hanging out face-to-face: Overlap in adolescents’ online and offline social networks. Dev Psychol, 48(2): 356–368. 10.1037/a0026980 22369341

[68] Einhorn H., & Hogarth R. 1981 Behavioral decision theory: Processes of judgment and choice. Annu Rev Psychol, 32: 52–88.

[69] Davies M. 1997 Belief persistence after evidential discrediting: The impact of generated versus provided explanations on the likelihood of discredited outcomes. J Exp Soc Psychol, 33: 561–578.

[70] Castellano C., Vilone D., & Vespignani A. 2003 Incomplete ordering of the voter model on small-world networks. Europhys Lett, 63(1): 153–158.

[71] Castellano C., Fortunato S., & Loreto V. 2009 Statistical physics of social dynamics. Rev Mod Phys, 81: 591–646.

[72] Miller J.H., & Page S.E. 2007 Complex Adaptive Systems. NJ: Princeton University Press.

[73] Ahn Y.-Y., Bagrow J.P., & Lehmann S. 2010 Link communities reveal multiscale complexity in networks. Nature, 466: 761–764. 10.1038/nature09182 20562860

[74] Weng L., Menczer F., & Ahn Y.-Y. 2013 Virality prediction and community structure in social networks. Sci Rep, 3: 2522 10.1038/srep02522 23982106 PMC3755286

[75] Podolny J. 1993 A status-based model of market competition. Am J Sociol, 98: 829–872.

[76] White H.C., Boorman S.A., & Breiger R.L. 1976 Social structure from multiple networks I: Block models of roles and positions. Am J Sociol, 87: 730–779.

[77] Burt R. 2007 Secondhand brokerage: Evidence on the importance of local structure for managers, bankers, and analysts. Acad Manage J, 50(1): 119–148.

[78] Kitsak M., Gallos L.K., Havlin S., Liljeros F., Muchnik L., Stanley H.E., & Makse H.A. 2010 Nat Phys, 6: 888–893.

[79] Pollet T.V., Roberts S.G.B., & Dunbar R.I.M. 2011 Extraverts have larger social network layers: But do not feel emotionally closer to individuals at any layer. J Individ Differ, 32(3): 161–169.

