# Supplementary material for: Social networks and inference about unknown events: A case of the match between Google’s AlphaGo and Sedol Lee
Source: PLoS One. 2017 Feb 21;12(2):e0171472. doi: 10.1371/journal.pone.0171472 (PMC5319654; doi:10.1371/journal.pone.0171472)
Supplement: S1 Text — (DOCX) [file pone.0171472.s002.docx]

**S1 Text**

The variables used for AlphaGo study.

The following are the name and definition of each variable.

- IRB ID, the participant identification number.
- Gender, 0 if female, 1 otherwise.
- Degree centrality, # of direct contacts that a person maintains.
- Ego network density, the number of indirect ties (i.e., ties among direct contacts) divided by the maximum possible number of indirect ties in a person’s ego network.
- High density group, 1 if ego network density >=0.5, 0 otherwise.
- Prior beliefs, each participant’s ex ante belief in the Go capability of Sedol Lee to win against AlphaGo which was measured in March. A six Likert scale.
- Outcome prediction, each person’s prediction about the winner of a given game. 0 if Sedol Lee wins, 1 otherwise.
- Accuracy rate in outcome prediction, the proportion of each person’s correct predictions about the game outcome.
- Being surprised by the result of the 1^st^ game (Result surprise), each person’s emotional response (being surprised) as to the outcome of the first game.
- Knowledge AI, each score of the three sub-items (a 9 Likert scale) as well as their average.
- Knowledge of Go, each score of the three sub-items (a 9 Likert scale) as well as their average.
- TV exposure, 1 if a person watched a given game and 0 otherwise.
- Other media exposure, 1 if a person read news or articles about a given game and 0 otherwise.
- Posterior belief, reversely coded, each participant’s ex post belief in the Go capability of Sedol Lee to win against AlphaGo which was measured in April. A five Likert scale.
- IPSAQ scores, 16 negative events, 16 positive events, the scores for positive events internal attribution, positive events personal attribution, positive events situational attribution, negative events internal attribution, negative events personal attribution, and negative events situational attribution, and the scores for externalizing bias and personalizing bias.
- IRI scores, the scores for overall, personal distress, perspective taking, empathic concern and fantasizing. Item 3, 4, 7, 12, 13, 14, 15, 18, and 19 are reverse-coded.

The variables used for Replication Tests.

The following are the name and definition of each variable.

- Cubee ID, the participant identification number.
- Impeachment, a 6 Likert scale, whether the parliament would vote to impeach the president by December 9, 2016 (6 being highly likely and 1 being highly unlikely).
- Voted before, 1 if I voted in the last general election in April 2016 and 2 otherwise.
- Sanders’ winning probability, the counterfactual assessment of the US presidential election, “the probability of Sanders winning the 2016 presidential election if he had been the candidate for the Democratic Party?”
- Sanders condition, 1 if the participant was primed with an article about Sanders; 2 otherwise.
- Degree centrality, # of direct contacts that a person maintains for one year.
- Ego network density, the number of indirect ties (i.e., ties among direct contacts) divided by the maximum possible number of indirect ties in a person’s ego network.
- KakaoTalk group-discussion rooms (#) that you were engaged in for last 10 days
- User of Facebook, 1 if yes; 2 otherwise.
- Alter 1 ~ Alter 5, “whether my offline friends in ego network are also facebook friends”, 1 if yes and 2 otherwise.

*Please note that for the prediction items that below, the on-line survey was designed in a way that the order of candidates was randomized for each participant*.

- Directing, the prediction of directing for Blue Dragon Film Festival on November 25, 2016.

(1) Kim, Jiwoon (2) Na, Hong-jin [**2016 Winner**] (3) Park, Chan-Wook, (4) Woo, Min-Ho,

(5) Lee, Joon-Ik

- Best Picture, the prediction of best picture

(1)The Wailing (2) Inside Men [**2016 Winner**] (3) Dongju: The Portrait of a Poet, (4) The Age of Shadows, (5) Train to Busan

- Actor in a supporting role

(1)Kim, Eui-Sung (2) Um, Tae-goo (3) Jun Kunimura [**2016 Winner**] (4) Ma, Dong-seok, (5) Oh, Dal-su

- Actress in a supporting role,
- (1)Ra, Mi-ran (2) Park, So-Dam [**2016 Winner**] (3) Bae, Doona (4) Jung, Yu-mi (5) Chun, Woo-hee
- At Cinema, # of watching films at the cinema for the last one year
- On the Phone, # of watching films on the phone for the last one month
- The last four columns refer to the subjective confidence in each participant’s prediction for a given category (6 Likert Scale)
