# Supplementary material for: Social networks and inference about unknown events: A case of the match between Google’s AlphaGo and Sedol Lee
Source: PLoS One. 2017 Feb 21;12(2):e0171472. doi: 10.1371/journal.pone.0171472 (PMC5319654; doi:10.1371/journal.pone.0171472)
Supplement: S2 Text — (DOCX) [file pone.0171472.s003.docx]

**S2 Text**

(1) The Composition of Participants

A total of 100 volunteers for multiple experiments were recruited for the research design in 2016. Each of these 100 volunteers was assigned a subject ID, namely, IRB ID, which begins from XJWR0025. For this study, a 63 of them in March agreed to participate. For our posterior belief survey in April, 7 of them were dropped from the sample. Owing to missing values on network density, more precisely, data on connections among direct contacts, the final sample for AlphaGo match prediction was 43 and that for posterior belief was 37. The details for the composition of the participants for this study are the following:

Participation in each survey (Yes= 1, No = 0, Missing = X)

|  | **Participation in each survey** | | |
| --- | --- | --- | --- |
|  | **January** | **March** | **April** |
| **IRB ID** | Network Survey | AlphaGo Study | Posterior Belief Survey |
| **XJWR0025** | 0 | 1 | 1 |
| **XJWR0026** | 0 | 1 | 1 |
| **XJWR0027** | 0 | 0 |  |
| **XJWR0028** | 0 | 1 | 1 |
| **XJWR0029** | 0 | 1 | 1 |
| **XJWR0030** | 0 | 1 | 1 |
| **XJWR0031** | 0 | 1 | 1 |
| **XJWR0032** | 0 | 1 | 1 |
| **XJWR0033** | 0 | 0 |  |
| **XJWR0034** | 0 | 1 | **0** |
| **XJWR0035** | 0 | 0 |  |
| **XJWR0036** | 0 | 0 |  |
| **XJWR0037** | 0 | 1 | 1 |
| **XJWR0038** | 0 | 1 | 1 |
| **XJWR0039** | 0 | 0 |  |
| **XJWR0040** | 0 | 1 | 1 |
| **XJWR0041** | 0 | 0 |  |
| **XJWR0042** | 0 | 1 | 1 |
| **XJWR0043** | 1 | 1 | **0** |
| **XJWR0044** | X | 1 | 1 |
| **XJWR0045** | X | 1 | 1 |
| **XJWR0046** | X | 1 | 1 |
| **XJWR0047** | 1 | 0 |  |
| **XJWR0048** | 1 | 1 | 1 |
| **XJWR0049** | 1 | 1 | **0** |
| **XJWR0050** | 1 | 0 |  |
| **XJWR0051** | 1 | 0 |  |
| **XJWR0052** | 1 | 1 | 1 |
| **XJWR0053** | 1 | 1 | 1 |
| **XJWR0054** | 1 | 0 |  |
| **XJWR0055** | 1 | 1 | 1 |
| **XJWR0056** | 1 | 0 |  |
| **XJWR0057** | 1 | 0 |  |
| **XJWR0058** | 1 | 1 | 1 |
| **XJWR0059** | 1 | 0 |  |
| **XJWR0060** | 1 | 1 | **0** |
| **XJWR0061** | 1 | 0 |  |
| **XJWR0062** | 1 | 1 | 1 |
| **XJWR0063** | 1 | 1 | 1 |
| **XJWR0064** | 1 | 1 | 1 |
| **XJWR0065** | 1 | 1 | 1 |
| **XJWR0066** | 1 | 1 | 1 |
| **XJWR0067** | 1 | 1 | 1 |
| **XJWR0068** | 1 | 1 | **0** |
| **XJWR0069** | 1 | 1 | 1 |
| **XJWR0070** | 1 | 1 | 1 |
| **XJWR0071** | 1 | 1 | 1 |
| **XJWR0072** | 1 | 1 | 1 |
| **XJWR0073** | 1 | 0 |  |
| **XJWR0074** | 1 | 1 | 1 |
| **XJWR0075** | 1 | 1 | 1 |
| **XJWR0076** | 1 | 1 | 1 |
| **XJWR0077** | 1 | 0 |  |
| **XJWR0078** | 1 | 0 |  |
| **XJWR0079** | 1 | 1 | 1 |
| **XJWR0080** | 1 | 0 |  |
| **XJWR0081** | 1 | 0 |  |
| **XJWR0082** | 1 | 1 | 1 |
| **XJWR0083** | 1 | 1 | 1 |
| **XJWR0084** | 1 | 1 | **0** |
| **XJWR0085** | 1 | 0 |  |
| **XJWR0086** | 1 | 0 |  |
| **XJWR0087** | 1 | 1 | 1 |
| **XJWR0088** | 1 | 1 | 1 |
| **XJWR0089** | 1 | 1 | 1 |
| **XJWR0090** | 1 | 0 |  |
| **XJWR0091** | 1 | 1 | 1 |
| **XJWR0092** | 1 | 1 | 1 |
| **XJWR0093** | 1 | 0 |  |
| **XJWR0094** | 1 | 0 |  |
| **XJWR0095** | 1 | 1 | **0** |
| **XJWR0096** | 1 | 0 |  |
| **XJWR0097** | 1 | 1 | 1 |
| **XJWR0098** | 1 | 1 | 1 |
| **XJWR0099** | X | 0 |  |
| **XJWR0100** | 1 | 1 | 1 |
| **XJWR0101** | 1 | 0 |  |
| **XJWR0102** | 1 | 0 |  |
| **XJWR0103** | 1 | 0 |  |
| **XJWR0104** | 1 | 0 |  |
| **XJWR0105** | 1 | 1 | 1 |
| **XJWR0106** | 1 | 1 | 1 |
| **XJWR0107** | 1 | 1 | 1 |
| **XJWR0108** | 1 | 1 | 1 |
| **XJWR0109** | 1 | 1 | 1 |
| **XJWR0110** | 1 | 0 |  |
| **XJWR0111** | 1 | 0 |  |
| **XJWR0112** | 0 | 0 |  |
| **XJWR0113** | 1 | 0 |  |
| **XJWR0114** | 1 | 1 | 1 |
| **XJWR0115** | 1 | 1 | 1 |
| **XJWR0116** | 1 | 0 |  |
| **XJWR0117** | 1 | 0 |  |
| **XJWR0118** | 1 | 1 | 1 |
| **XJWR0119** | 1 | 0 |  |
| **XJWR0120** | 0 | 1 | 1 |
| **XJWR0121** | 0 | 1 | 1 |
| **XJWR0122** | 0 | 1 | 1 |
| **XJWR0123** | 0 | 1 | 1 |
| **XJWR0124** | 0 | 1 | 1 |
|  |  |  |  |
| **# of participants in each survey** | 76 | 63 | 56 |
| **# of participants in Jan & March** |  | 46 |  |
| **# of participants in Jan & April** |  |  | 40 |
| **Sample with no missing values** | **72** | **43** | **37** |

Please note that gray-colored entries in the fourth column indicate those who did not participate in the AlphaGo survey.

(1-1) The Composition of Participants for Replication Tests (IRB no. 1604/003-014)

A total of 136 volunteers for multiple experiments had been recruited since May 2016. Each of these 136 volunteers was assigned a subject ID, which begins from CUBEE0001. For the survey of social relations, 95 participated from October 31, 2016 to November 18, 2016. A total of 84 participants also participated in replication tests that were run on November 25, 2016. Owing to missing values on network density, a total of 79 observations were obtained for each measure. The details for the composition of the participants are the following:

Participation in each survey (Yes= 1, No = 0); Ego density not defined (i.e., the size of network is one)= (X)

|  | **Participation in each survey** | |
| --- | --- | --- |
|  | **October & November** | **November** |
| **CUBEE ID** | Social Network Survey | Replication Test |
| **CUBEE0001** | 1 | 1 |
| **CUBEE0002** | 1 | 1 |
| **CUBEE0003** | 1 | 1 |
| **CUBEE0004** | 1 | 1 |
| **CUBEE0005** | 1 | 1 |
| **CUBEE0007** | 1 | 1 |
| **CUBEE0008** | 1 | 1 |
| **CUBEE0009** | 1 | 1 |
| **CUBEE0010** | 1 | 1 |
| **CUBEE0011** | 1 | 1 |
| **CUBEE0012** | 1 | 1 |
| **CUBEE0014** | 1 | 1 |
| **CUBEE0015** | 1 | 1 |
| **CUBEE0016** | X | 1 |
| **CUBEE0018** | 1 | 1 |
| **CUBEE0019** | 1 | 1 |
| **CUBEE0021** | 1 | 0 |
| **CUBEE0022** | X | 1 |
| **CUBEE0024** | X | 1 |
| **CUBEE0025** | 1 | 1 |
| **CUBEE0027** | 1 | 1 |
| **CUBEE0030** | 1 | 1 |
| **CUBEE0032** | 1 | 1 |
| **CUBEE0033** | 1 | 1 |
| **CUBEE0036** | 1 | 1 |
| **CUBEE0037** | 1 | 1 |
| **CUBEE0038** | 1 | 0 |
| **CUBEE0039** | 1 | 0 |
| **CUBEE0040** | 1 | 1 |
| **CUBEE0041** | 1 | 1 |
| **CUBEE0045** | 1 | 1 |
| **CUBEE0046** | 1 | 0 |
| **CUBEE0047** | 1 | 1 |
| **CUBEE0048** | 1 | 1 |
| **CUBEE0050** | 1 | 0 |
| **CUBEE0051** | 1 | 1 |
| **CUBEE0052** | 1 | 1 |
| **CUBEE0053** | 1 | 1 |
| **CUBEE0054** | 1 | 1 |
| **CUBEE0058** | 1 | 1 |
| **CUBEE0059** | X | 1 |
| **CUBEE0060** | 1 | 1 |
| **CUBEE0062** | X | 0 |
| **CUBEE0063** | 1 | 1 |
| **CUBEE0064** | 1 | 1 |
| **CUBEE0065** | 1 | 1 |
| **CUBEE0067** | 1 | 1 |
| **CUBEE0068** | 1 | 1 |
| **CUBEE0072** | 1 | 1 |
| **CUBEE0073** | 1 | 1 |
| **CUBEE0074** | 1 | 1 |
| **CUBEE0075** | 1 | 1 |
| **CUBEE0076** | 1 | 1 |
| **CUBEE0077** | 1 | 1 |
| **CUBEE0078** | 1 | 1 |
| **CUBEE0080** | 1 | 1 |
| **CUBEE0081** | 1 | 1 |
| **CUBEE0085** | 1 | 1 |
| **CUBEE0086** | 1 | 1 |
| **CUBEE0087** | 1 | 0 |
| **CUBEE0088** | 1 | 0 |
| **CUBEE0090** | 1 | 1 |
| **CUBEE0091** | 1 | 1 |
| **CUBEE0094** | 1 | 1 |
| **CUBEE0095** | 1 | 1 |
| **CUBEE0096** | X | 1 |
| **CUBEE0099** | 1 | 1 |
| **CUBEE0100** | 1 | 1 |
| **CUBEE0101** | 1 | 1 |
| **CUBEE0103** | 1 | 0 |
| **CUBEE0104** | 1 | 1 |
| **CUBEE0105** | 1 | 1 |
| **CUBEE0107** | 1 | 1 |
| **CUBEE0109** | 1 | 1 |
| **CUBEE0110** | 1 | 1 |
| **CUBEE0113** | 1 | 1 |
| **CUBEE0114** | 1 | 0 |
| **CUBEE0115** | 1 | 1 |
| **CUBEE0116** | 1 | 1 |
| **CUBEE0117** | 1 | 1 |
| **CUBEE0118** | 1 | 1 |
| **CUBEE0119** | 1 | 1 |
| **CUBEE0121** | 1 | 1 |
| **CUBEE0122** | 1 | 1 |
| **CUBEE0123** | 1 | 0 |
| **CUBEE0124** | 1 | 1 |
| **CUBEE0126** | 1 | 1 |
| **CUBEE0128** | 1 | 1 |
| **CUBEE0129** | 1 | 1 |
| **CUBEE0131** | 1 | 1 |
| **CUBEE0132** | 1 | 1 |
| **CUBEE0133** | 1 | 1 |
| **CUBEE0134** | 1 | 1 |
| **CUBEE0135** | 1 | 1 |
| **CUBEE0136** | 1 | 1 |
| **# of participants in each survey** | 95 | 84 |
| **Sample with no missing values on ego network density** | 89 | 79 |

(2) **Fig 3**.

| Lee(*p*) | Those who predicted Lee's winning in a given game (proportion) | | | | | |  |
| --- | --- | --- | --- | --- | --- | --- | --- |
| Alpha(*p*) | Those who predicted Alphago's winning in a given game (proportion) | | | | | |  |
|  |  |  |  |  |  |  |  |
| Total | ***N*=63** |  | **Game 1** | **Game 2** | **Game 3** | **Game 4** | **Game 5** |
|  | Lee(*p*) | Proportion | 0.793651 | 0.603175 | 0.269841 | 0.079365 | 0.349206 |
|  |  | # of respondents | 50 | 38 | 17 | 5 | 22 |
|  | AlphaGo(*p*) | Proportion | 0.206349 | 0.396825 | 0.730159 | 0.920635 | 0.650794 |
|  |  | # of respondents | 13 | 25 | 46 | 58 | 41 |
|  | **Odds Ratio** | Lee(p)/AlphaGo(p) | 3.846154 | 1.52 | 0.369565 | 0.086207 | 0.536585 |
|  |  |  |  |  |  |  |  |
| High Prior Group | ***N*=52** |  | **Game 1** | **Game 2** | **Game 3** | **Game 4** | **Game 5** |
|  | Lee(*p*) | Proportion | 0.826923 | 0.673077 | 0.326923 | 0.096154 | 0.307692 |
|  |  | # of respondents | 43 | 35 | 17 | 5 | 16 |
|  | AlphaGo(*p*) | Proportion | 0.173077 | 0.326923 | 0.673077 | 0.903846 | 0.692308 |
|  |  | # of respondents | 9 | 17 | 35 | 47 | 36 |
|  | **Odds Ratio** | Lee(p)/AlphaGo(p) | 4.777778 | 2.058824 | 0.485714 | 0.106383 | 0.444444 |
|  |  |  |  |  |  |  |  |
| Low Prior Group | ***N*=11** |  | **Game 1** | **Game 2** | **Game 3** | **Game 4** | **Game 5** |
|  | Lee(*p*) | Proportion | 0.636364 | 0.272727 | 0 | 0 | 0.545455 |
|  |  | # of respondents | 7 | 3 | 0 | 0 | 6 |
|  | AlphaGo(*p*) | Proportion | 0.363636 | 0.727273 | 1 | 1 | 0.454545 |
|  |  | # of respondents | 4 | 8 | 11 | 11 | 5 |
|  | **Odds Ratio** | Lee(p)/AlphaGo(p) | 1.75 | 0.375 | 0 | 0 | 1.2 |

(3) **Fig 4 (Sas proc ttest)**. Confidence interval was computed as follows.

| **Group** | ***N*** | **Mean** | **Std Dev** | **Std Err** | **Minimum** | **Maximum** |  |
| --- | --- | --- | --- | --- | --- | --- | --- |
| **Low Prior** | 11 | 0.6364 | 0.5045 | 0.1521 | 0 | 1 |  |
| **High Prior** | 52 | 0.1731 | 0.382 | 0.053 | 0 | 1 |  |
| **Diff (1-2)** |  | 0.4633 | 0.4046 | 0.1343 |  |  |  |
|  |  |  |  |  |  |  |  |
| **Group** | **Method** | **Mean** | **95% CL Mean** | | **Std Dev** | **95% CL Std Dev** | |
| **Low Prior** |  | 0.6364 | 0.2974 | 0.9753 | 0.5045 | 0.3525 | 0.8854 |
| **High Prior** |  | 0.1731 | 0.0667 | 0.2794 | 0.382 | 0.3201 | 0.4737 |
| **Diff (1-2)** | **Pooled** | 0.4633 | 0.1948 | 0.7318 | 0.4046 | 0.3438 | 0.4917 |
| **Diff (1-2)** | **Satterthwaite** | 0.4633 | 0.114 | 0.8126 |  |  |  |
|  |  |  |  |  |  |  |  |
| **Method** | **Variances** | **DF** | **t Value** | **Pr > \|t\|** |  |  |  |
| **Pooled** | Equal | 61 | 3.45 | 0.001 |  |  |  |
| **Satterthwaite** | Unequal | 12.536 | 2.88 | 0.0134 |  |  |  |
| **Cochran** | Unequal | . | 2.88 | 0.0153 |  |  |  |

(4) **Fig 5**.

| Lee(*p*) | Those who predicted Lee's winning in a given game (proportion) | | | | | |  |
| --- | --- | --- | --- | --- | --- | --- | --- |
| Alpha(*p*) | Those who predicted Alphago's winning in a given game (proportion) | | | | | |  |
|  |  |  |  |  |  |  |  |
| Low Density Group | ***N*=26** |  | **Game 1** | **Game 2** | **Game 3** | **Game 4** | **Game 5** |
|  | Lee(*p*) | Proportion | 0.692308 | 0.5 | 0.307692 | 0.076923 | 0.307692 |
|  |  | # of respondents | 18 | 13 | 8 | 2 | 8 |
|  | AlphaGo(*p*) | Proportion | 0.307692 | 0.5 | 0.692308 | 0.923077 | 0.692308 |
|  |  | # of respondents | 8 | 13 | 18 | 24 | 18 |
|  | **Odds Ratio** | Lee(p)/AlphaGo(p) | 2.25 | 1 | 0.444444 | 0.083333 | 0.444444 |
|  |  |  |  |  |  |  |  |
| High Density Group | ***N*=17** |  | **Game 1** | **Game 2** | **Game 3** | **Game 4** | **Game 5** |
|  | Lee(*p*) | Proportion | 0.882353 | 0.588235 | 0.294118 | 0.117647 | 0.411765 |
|  |  | # of respondents | 15 | 10 | 5 | 2 | 7 |
|  | AlphaGo(*p*) | Proportion | 0.117647 | 0.411765 | 0.705882 | 0.882353 | 0.588235 |
|  |  | # of respondents | 2 | 7 | 12 | 15 | 10 |
|  | **Odds Ratio** | Lee(p)/AlphaGo(p) | 7.5 | 1.428571 | 0.416667 | 0.133333 | 0.7 |

(5) **Fig 6 (Sas proc ttest)**. Confidence interval was computed as follows.

| **Group** | ***N*** | **Mean** | **Std Dev** | **Std Err** | **Minimum** | **Maximum** |  |
| --- | --- | --- | --- | --- | --- | --- | --- |
| **Low Density** | 26 | 0.3846 | 0.4961 | 0.0973 | 0 | 1 |  |
| **High Density** | 17 | 0.1176 | 0.3321 | 0.0805 | 0 | 1 |  |
| **Diff (1-2)** |  | 0.267 | 0.4395 | 0.1371 |  |  |  |
|  |  |  |  |  |  |  |  |
| **Group** | **Method** | **Mean** | **95% CL Mean** | | **Std Dev** | **95% CL Std Dev** | |
| **Low Density** |  | 0.3846 | 0.1842 | 0.585 | 0.4961 | 0.3891 | 0.6849 |
| **High Density** |  | 0.1176 | -0.0531 | 0.2884 | 0.3321 | 0.2473 | 0.5054 |
| **Diff (1-2)** | **Pooled** | 0.267 | -0.00986 | 0.5438 | 0.4395 | 0.3616 | 0.5604 |
| **Diff (1-2)** | **Satterthwaite** | 0.267 | 0.0119 | 0.5221 |  |  |  |
|  |  |  |  |  |  |  |  |
| **Method** | **Variances** | **DF** | **t Value** | **Pr > \|t\|** |  |  |  |
| **Pooled** | Equal | 41 | 1.95 | 0.0583 |  |  |  |
| **Satterthwaite** | Unequal | 40.954 | 2.11 | 0.0407 |  |  |  |
| **Cochran** | Unequal | . | 2.11 | 0.0471 |  |  |  |

(6) **Fig 7 (Sas proc ttest)**.

| Small Sized Network | **Group** | ***N*** | **Mean** | **Std Dev** | **Std Err** | **Minimum** | **Maximum** |  |
| --- | --- | --- | --- | --- | --- | --- | --- | --- |
|  | **Low Density** | 13 | 0.5385 | 0.2631 | 0.073 | 0 | 0.8 |  |
|  | **High Density** | 11 | 0.3636 | 0.1748 | 0.0527 | 0 | 0.6 |  |
|  | **Diff (1-2)** |  | 0.1748 | 0.2273 | 0.0931 |  |  |  |
|  |  |  |  |  |  |  |  |  |
|  | **Group** | **Method** | **Mean** | **95% CL Mean** | | **Std Dev** | **95% CL Std Dev** | |
|  | **Low Density** |  | 0.5385 | 0.3795 | 0.6975 | 0.2631 | 0.1887 | 0.4343 |
|  | **High Density** |  | 0.3636 | 0.2462 | 0.4811 | 0.1748 | 0.1221 | 0.3067 |
|  | **Diff (1-2)** | **Pooled** | 0.1748 | -0.0183 | 0.3679 | 0.2273 | 0.1758 | 0.3217 |
|  | **Diff (1-2)** | **Satterthwaite** | 0.1748 | -0.0124 | 0.362 |  |  |  |
|  |  |  |  |  |  |  |  |  |
|  | **Method** | **Variances** | **DF** | **t Value** | **Pr > \|t\|** |  |  |  |
|  | **Pooled** | Equal | 22 | 1.88 | 0.0737 |  |  |  |
|  | **Satterthwaite** | Unequal | 20.944 | 1.94 | 0.0657 |  |  |  |
|  | **Cochran** | Unequal | . | 1.94 | 0.0776 |  |  |  |
|  |  |  |  |  |  |  |  |  |
|  |  |  |  |  |  |  |  |  |
| Large Sized Network | **Group** | ***N*** | **Mean** | **Std Dev** | **Std Err** | **Minimum** | **Maximum** |  |
|  | **Low Density** | 13 | 0.3692 | 0.1797 | 0.0499 | 0 | 0.6 |  |
|  | **High Density** | 6 | 0.4333 | 0.1966 | 0.0803 | 0.2 | 0.6 |  |
|  | **Diff (1-2)** |  | -0.0641 | 0.1849 | 0.0912 |  |  |  |
|  |  |  |  |  |  |  |  |  |
|  | **Group** | **Method** | **Mean** | **95% CL Mean** | | **Std Dev** | **95% CL Std Dev** | |
|  | **Low Density** |  | 0.3692 | 0.2606 | 0.4778 | 0.1797 | 0.1289 | 0.2967 |
|  | **High Density** |  | 0.4333 | 0.227 | 0.6397 | 0.1966 | 0.1227 | 0.4823 |
|  | **Diff (1-2)** | **Pooled** | -0.0641 | -0.2566 | 0.1284 | 0.1849 | 0.1387 | 0.2772 |
|  | **Diff (1-2)** | **Satterthwaite** | -0.0641 | -0.2777 | 0.1495 |  |  |  |
|  |  |  |  |  |  |  |  |  |
|  | **Method** | **Variances** | **DF** | **t Value** | **Pr > \|t\|** |  |  |  |
|  | **Pooled** | Equal | 17 | -0.7 | 0.4918 |  |  |  |
|  | **Satterthwaite** | Unequal | 9.0398 | -0.68 | 0.5145 |  |  |  |
|  | **Cochran** | Unequal | . | -0.68 | 0.5231 |  |  |  |

(7) **Fig 8**.

| **Posterior Belief** | |  |  |  |  |  |
| --- | --- | --- | --- | --- | --- | --- |
| ***Group*** | *N* | (1) | (2) | (3) | (4) | (5) |
| Low Density | 22 | 0 | 0 | 0.182 | 0.5 | 0.318 |
| High Density | 15 | 0 | 0 | 0.2 | 0.6 | 0.2 |
|  |  |  |  |  |  |  |

(8) **Fig 9 (Spss)**.

| **Group** | **N** | **Mean** | **Std Dev** | **Std Err** | **Minimum** | **Maximum** |
| --- | --- | --- | --- | --- | --- | --- |
| **Low Density** | 22 | 2.6691 | 0.75439 | 0.16084 | 1.29 | 3.71 |
| **High Density** | 13 | 3.1977 | 0.74073 | 0.20544 | 2.14 | 4.57 |
| **Diff (1-2)** |  | -0.5286 | 0.01366 | -0.0446 |  |  |
|  |  |  |  |  |  |  |
| **Group** | **Variances** | **Mean** | **95% CL Mean** | | **t value** | **Pr > \|t\|** |
| **Diff (1-2)** | **Equal** | -0.5286 | -1.06201 | 0.0048 | -2.016 | 0.052 |
| **Diff (1-2)** | **Unequal** | -0.5286 | -1.06522 | 0.00801 | -2.026 | 0.053 |
|  |  |  |  |  |  |  |
|  | **Levene's test** |  | **F Value** | **Pr > \|t\|** |  |  |
|  | Equal variance |  | 0.061 | 0.806 |  |  |

(9) **Means, standard deviations, and correlations among variables**.

|  | *N* | Mean | S. D. | 2 | 3 | 4 |
| --- | --- | --- | --- | --- | --- | --- |
| 1. Ego network density | 43 | 0.486 | 0.272 | 0.859**^†^** | -0.435**^†^** | -0.012 |
| 2. High network density (1 if yes) | 43 | 0.395 | 0.495 |  | -0.286**^†^** | -0.019 |
| 3. Degree centrality | 43 | 5.651 | 2.369 |  |  | 0.077 |
| 4. Prior beliefs | 43 | 3.674 | 1.189 |  |  |  |
| 5. Posterior beliefs | 37 | 4.081 | 0.682 |  |  |  |
| 6. Deviant prediction in the 1^st^ game | 43 | 0.279 | 0.454 |  |  |  |
| 7. Accuracy rate in outcome prediction | 43 | 0.428 | 0.216 |  |  |  |
| 8. Gender (1 if male) | 43 | 0.628 | 0.489 |  |  |  |
| 9. Externalizing bias | 37 | 2.162 | 5.069 |  |  |  |
| 10. Personal Distress | 35 | 2.865 | 0.783 |  |  |  |

|  | 5 | 6 | 7 | 8 | 9 | 10 |
| --- | --- | --- | --- | --- | --- | --- |
| 1. Ego network density | -0.141 | -0.236 | -0.238 | 0.076 | -0.215 | 0.149 |
| 2. High network density (dummy = 1) | -0.099 | -0.291**^†^** | -0.150 | 0.032 | -0.313**^†^** | 0.331**^†^** |
| 3. Degree centrality | -0.100 | 0.137 | 0.038 | 0.049 | 0.061 | 0.023 |
| 4. Prior beliefs | -0.197 | -0.445**^†^** | -0.315**^†^** | 0.155 | -0.198 | -0.247 |
| 5. Posterior beliefs |  | 0.185 | 0.333**^†^** | 0.011 | -0.205 | -0.081 ^a^ |
| 6. Deviant prediction in the 1^st^ game |  |  | 0.209 | -0.057 | 0.074 | 0.152 |
| 7. Accuracy rate in outcome prediction |  |  |  | -0.305**^†^** | 0.149 | 0.018 |
| 8. Gender (1 if male) |  |  |  |  | -0.119 | -0.322**^†^** |
| 9. Externalizing bias |  |  |  |  |  | 0.053 ^a^ |
| 10. Personal distress |  |  |  |  |  |  |

^a^ *N* = 33; **^†^** *p* <.10

(10) **Replication Tests**

10.1. Power Analysis for the Tests: we designed the replication tests according to the following scenarios for power analysis. On the basis of the AlphaGo study, we expected that the high density group were poor at the prediction of film wards (i.e., 0.20), whereas the prediction by the low density group would yield a difference in proportions of 0.30. For the prediction of the President impeachment, we expected that the proportion of the high prospect for the impeachment in the high density group would be as high as the majority view (79.5% in support of the impeachment according to a Realmeter on the November 24, 2016: <http://www.realmeter.net>), whereas the low density group would hold a balanced view (i.e., 50%). Note that we drew on Cohen (1992) and tried to keep the effect size to be moderate. Fisher’s exact test indicated that under various contingencies for our prediction tasks, the sample size per density group to achieve the desired power of 80% at the 5% one-sided level of significance was 37. At the 10% one-sided level of significance it was 29. Accordingly, we tried to obtain 80 observations, which were 40 per the density group.

Reference: Cohen, J. 1992. A power primer. *Psychological Bulletin*, 112(1): 155-159.

10.2. Characteristics of Persons whose Ego Network Size is Two (*N*=7):

|  | **Cubee ID** | **KakaoTalk Group-Chatroom** | **At the Cinema(#)** | **On the Phone (#)** |
| --- | --- | --- | --- | --- |
|  | Cubee0003 | 12 | 10 | 2 |
|  | Cubbee0032 | 12 | 4 | 2 |
|  | Cubee0053 | 16 | 1 | 0 |
|  | Cubee0073 | 8 | 40 | 10 |
|  | Cubee0095 | 9 | 7 | 10 |
|  | Cubee0104 | 16 | 4 | 2 |
|  | Cubee0126 | 13 | 12 | 1 |
|  | Average | 12.29 | 11.14 | 3.86 |

10.3. Table 3 and Inconsistent Responses for Facebook Users:

In the replication tests, we examined the online and offline overlap in social contacts from the survey of whether significant others in one’s social relations are also friends in Facebook. Among those who reported that they were not Facebook users, eight persons indicated that some of their social contacts were also Facebook friends. Besides coding error, one possible reason for this would be that the respondents at the time of survey were not using Facebook yet their social contacts used to be Facebook friends in the past. For the computation of Table 3, we did not include these 8 observations for on/offline overlap in social contacts.

10.4. Means, standard deviations, and correlations among variables.

|  | *N* | Mean | S. D. | 2 | 3 | 4 |
| --- | --- | --- | --- | --- | --- | --- |
| 1. Ego network density | 79 | 0.333 | 0.275 | 0.731**^†^** | -0.184 | 0.004 |
| 2. Middle+High Density Group (1 if yes) | 79 | 0.443 | 0.499 |  | -0.191**^†^** | 0.157 |
| 3. Correct prediction of best picture (1 if yes) | 79 | 0.329 | 0.473 |  |  | 0.039 |
| 4. High prospects for impeachment (1 if yes) | 79 | 0.785 | 0.414 |  |  |  |
| 5. Sanders’ winning probability (%) | 79 | 46.38 | 22.57 |  |  |  |
| 6. Sanders’ scenario (1 if yeas) | 79 | 0.468 | 0.502 |  |  |  |

|  | 5 | 6 |
| --- | --- | --- |
| 1. Ego network density | -0.086 | 0.084 |
| 2. Middle+High Density Group (1 if yes) | -0.042 | 0.133 |
| 3. Correct prediction of best picture (1 if yes) | -0.061 | -0.009 |
| 4. High prospects for impeachment (1 if yes) | 0.073 | 0.059 |
| 5. Sanders’ winning probability (%) |  | 0.077 |
| 6. Sanders’ scenario (1 if yeas) |  |  |

**^†^** *p* <.10

10.5. Prediction of Artworks:

|  |  | **“Are you confident in your prediction?”(6 Likert Scale)** (mean, s.d.) | | |
| --- | --- | --- | --- | --- |
|  | **Award Category** | **Sample Average** | **Low Density (*N*=44)** | **Middle+High Density (*N*=35)** |
|  | Directing | 2.76(1.13) | 2.84(1.16) | 2.66(1.11) |
|  | Best Picture | 3.00(1.17) | 3.02(1.21) | 2.97(1.12) |
|  | Actress, supporting | 2.84(1.24) | 2.84(1.27) | 2.86(1.22) |
|  | Actor, supporting | 2.96(1.32) | 2.93(1.28) | 3.00(1.37) |

|  |  | **The Prediction of Best Picture Award** (frequency) | |
| --- | --- | --- | --- |
|  | **Films (**decreasing order of sales share 2016^*^) | **Low Density (*N*=44)** | **Middle+High Density (*N*=35)** |
|  | Train to Busan | 0.05 | 0.06 |
|  | The Age of Shadows | 0.09 | 0.06 |
|  | The Wailing | 0.34 | 0.29 |
|  | Inside Men**(**winner of best picture**) | **0.41** | 0.23 |
|  | Dongju: The Portrait of a Poet | 0.11 | **0.37** |

^*^ source: Korean Film Council:

(<http://www.kobis.or.kr/kobis/business/stat/offc/findYearlyBoxOfficeList.do?loadEnd=0&searchType=search&sSearchYearFrom=2016&sMultiMovieYn=&sRepNationCd>=)

|  | **Chi-Square Test for the Prediction of Best Picture Award** | | | |
| --- | --- | --- | --- | --- |
|  | **Prediction** | **Low Density** | **Middle+High Density** | **Total** |
|  | Incorrect | 26 | 27(77.1%) | 53 |
|  | Correct | 18(40.9%) | 8 | 26 |
|  | Total | 44(100%) | 35(100%) | 79 |
|  | Pearson Chi squared (df = 1) | 2.877, *p* = 0.090 (2-sided) | | |

|  | **Logit Model for the Prediction of Best Picture Award (*N*=72)** | | | |
| --- | --- | --- | --- | --- |
|  | **Parameter** | **Estimate (s.e.)** | **Chi-squared (df)** | **Pr > Chi-squared** |
|  | Intercept | 0.1132(0.4521) | 0.0627 | 0.8023 |
|  | Ego Network Density | -2.726(1.3802) | 3.9006 | 0.0483 |
|  | Model Fit Statistic | LR Chi-squared (1)= 4.8923., *p* = 0.027 (2-sided) | | |

10.6. Prediction of the President Impeachment:

|  | **Chi-Square Test for the Prediction of the Impeachment** | | | |
| --- | --- | --- | --- | --- |
|  | **High Prospect** | **Low Density** | **Middle+High Density** | **Total** |
|  | No | 12(27.3%) | 5(14.3%) | 17 |
|  | Yes | 32(72.7%) | 30(85.7%) | 62 |
|  | Total | 44(100%) | 35(100%) | 79 |
|  | Pearson Chi squared (df = 1) | 1.9468, *p* = 0.1629 (2-sided) | | |
|  |  |  | | |

|  | **Gallup Korea Poll on December 9 2016 (Gallup Korea Daily Opinion, #239)**  It was surveyed from December 6 to December 8 for a sample of 1012 Koreans | |
| --- | --- | --- |
|  | In Support of Impeachment | 81% (for the full sample) |
|  | In Support of Impeachment | 93% (for the respondents aged from 19 to 29) |

|  | **Logit Model for the Prediction of the Impeachment (*N*=79)** | | | |
| --- | --- | --- | --- | --- |
|  | **Parameter** | **Estimate (s.e.)** | **Chi-squared (df)** | **Pr > Chi-squared** |
|  | Intercept | 0.5680(0.5576) | 1.0377 | 0.3083 |
|  | Ego Network Density | 5.3403(3.1554) | 2.8644 | 0.0906 |
|  | Ego Network Density^2^ | -5.3480(3.0138) | 3.1489 | 0.0760 |
|  | Model Fit Statistic | LR Chi-squared (2)= 3.1441., *p* = 0.2076 (2-sided) | | |

10.7. Prediction of the US Presidential Election:

|  | **The Counterfactual Assessment of the Impeachment (*N*=79)** | | | |
| --- | --- | --- | --- | --- |
|  | **Condition** | **Winning Probability (mean)** | **s.d.** | **Max** |
|  | Clinton (*N* =42) | 44.7619 | 25.2053 | 85 |
|  | Snaders (*N*=37) | 48.2162 | 19.3407 | 80 |

|  | **A *t* Test for Group Difference in Logged Probability (*N*=77)** | | |
| --- | --- | --- | --- |
|  | **Condition** | **Winning Probability (mean)** | **s.d.** |
|  | A: Clinton (*N* =41) | 3.6138 | 0.7470 |
|  | B: Snaders (*N*=36) | 3.8294 | 0.4078 |
|  | Diff(A-B) | -0.2156 | 0.6126 |
|  | Satterthwaite Method (unequal variances) | *t* = -1.60 | *p* = 0.05765 |
|  | Equality of Variances (Folded F) | *F* (40,35)=3.36 | *p* = 0.0004 |

|  | **A *t* Test for Group Difference in Logged Probability (**the middle density group excluded**)** | | |
| --- | --- | --- | --- |
|  | **Condition** | **Winning Probability (mean)** | **s.d.** |
|  | A: Clinton (*N* =33) | 3.6058 | 0.7026 |
|  | B: Snaders (*N*=29) | 3.8446 | 0.4022 |
|  | Diff(A-B) | -0.2388 | 0.5821 |
|  | Satterthwaite Method (unequal variances) | *t* = -1.67 | *p* = 0.0529 |
|  | Equality of Variances (Folded F) | *F* (32,28)=3.05 | *p* = 0.0036 |

10.8. **The raw data for Fig 10**:

|  | **Fig 10** | | | |
| --- | --- | --- | --- | --- |
|  | **Bar #** | **Item** | **Average** | ***N*** |
|  | 1 | Support for impeachment (A Gallup Korea Poll released on December 9, 2016  for a cohort of aged from 19 to 29)(Daily Opinion # 239) | 0.930 | 161 |
|  | 2 | High prospects for impeachment by the middle and high density group | 0.857 | 35 |
|  | 3 | High prospect for impeachment by the low density group | 0.727 | 44 |

(11) **Questionnaires**.

1. Social network survey:

1) We often receive help from others or go for help on our study and social activities. When you work at your current institution, to whom you normally go for help and advice on work-related topics? In what below, please list either first names or initials of those to whom you go for help and advice.

1.1) From the listed persons, please identify persons who are friends or who know of each other well.

2. Emotional response to the 1^st^ Game (via google forms): What do you think of the result of the game?

1= highly expected; 9= highly surprising (9-point horizontal scale)

| ----1---- | ----2---- | ----3---- | ----4---- | ----5---- | ----6---- | ----7---- | ----8---- | ----9---- |
| --- | --- | --- | --- | --- | --- | --- | --- | --- |
|  |  |  |  | Neither surprising nor expected |  |  |  |  |

3. Media exposure:

1) TX exposure (via google forms): Have you watched the Go game between AlphaGo and Sedol Lee today?

🗌 Yes

🗌 No

2) Other media exposure (via google forms): Have you read news or articles about the Go game between AlphaGo and Sedol Lee today?

🗌 Yes

🗌 No

4. Knowledge of Go (via offline questionnaire)(Cronbach’s Alpha = 0.835):

1) I regularly read or heard of things about Go game.

2) I am familiar with the rule of Go game.

3) I am interested in Go game.

| ----1---- | ----2---- | ----3---- | ----4---- | ----5---- | ----6---- | ----7---- | ----8---- | ----9---- |
| --- | --- | --- | --- | --- | --- | --- | --- | --- |
| Strongly disagree |  |  |  | 보통 |  |  |  | Strongly agree |

5. Knowledge of AI (via offline questionnaire) (Cronbach’s Alpha = 0.855):

1) I regularly read or heard of things about artificial intelligence.

2) I am familiar with the mechanism of artificial intelligence.

3) I am interested in artificial intelligence.

| ----1---- | ----2---- | ----3---- | ----4---- | ----5---- | ----6---- | ----7---- | ----8---- | ----9---- |
| --- | --- | --- | --- | --- | --- | --- | --- | --- |
| Strongly disagree |  |  |  | 보통 |  |  |  | Strongly agree |

6. Internal, Personal and Situational Attributions Questionnaire (IPSAQ) / via offline questionnaire (take-home):

A person’s attribution style was measured by a Korean version of the IPSAQ (Kinderman & Bentall, 1996). Four Korean researchers participated in the forward-backward translation of the original version of the IPSAQ. They were MW Chon (MD) from Asan Medical Center, JW Hur (PhD) from Chung-Ang University, S Kwak (PhD candidate) from Seoul National University and J Seol (PhD candidate, bilingual) from Seoul National University.

7. Interpersonal Reactivity Index (IRI) / via offline questionnaire in the experimental room:

A person’s empathy for others was measured by the Interpersonal Reactivity Index (Davis, 1983). The Korean version of the IRI was based on Kang et al. (2009).

**Ref**. Kang, I., Kee, S., Kim, S. E., Jeong, B., Hwang, J. H., Song, J. E., & Kim, J. W. 2009. Reliability and validity of the Korean-version of Interpersonal Reactivity Index. *Journal of Korean Neuropsychiatric Association*, 48(5): 352-358.

8. Test of Politics (the counterfactual assessment of the US presidential election).

(1) The article for the Clinton scenario:

Donald Trump, president-elect, was outpaced by Hilary Clinton in the national popular vote and yet won the Electoral College vote by a large margin. Al Gore, the Democratic candidate, also won the popular vote yet lost in the Electoral College 16 years ago. According to New York Times on November 16 Hilary Clinton, former Secretary of State, in her first public remarks since conceding to President-elect Donald Trump, told the crowd, “I know that over the past week, a lot of people have asked themselves whether America was the country we thought it was” and added, “believe in our country, fight for our values and never give up”. She also said, “I believe the measure of any society is how we treat our children, and as we move forward into a new and in many ways uncertain future, that must be the test for America and ourselves.” “No child should be afraid to go to school because they’re Latino, or African-American, or Muslim, or because they have a disability.”

(2) The article for the Sanders scenario:

Senator Sanders (Democratic Party) stresses the Democratic Party to be transformed while leaving open the possibility of running again for president next time around. In a phone interview with Associated Press on November 10, Senator Sanders who lost the Democratic nomination for president in the 2016 election, said, “Four years is a long time from now” and added “We'll take one thing at a time, but I'm not ruling out anything.” He apparently prepares to win senate re-election in 2018 and may re-run for president in 2020. During the 2016 Democratic Party presidential primaries and caucuses, Senator Sanders, an independent during 30 years in congress, out-competed Clinton in a hypothetical election match-up against Donald Trump. Like Donald Trump, Bernie Sanders is outside of Washington, which distinguishes himself from Hillary Clinton who has a negative impression of an established politician. During primaries and caucuses, he was therefore able to take advantage of Donald Trump whose political advantage also comes from being an outsider politician.

**Naver Search Query**

This file illustrates the variation in the public exposure of the internet users in Korea to the Go-match between AlphaGo and Sedol Lees in 2016. The frequency of "Naver" search queries may reflect a person’s exposure to either AlphaGo or Sedol Lee at least in the online community. Note that "Naver" is the number 1 service provider in the domain of search engine in Korea in 2016 and that in Korea it is more popular than Google search engine. The following graph is based on the history of search queries in Naver portal as well as Naver shopping for a given period. We tracked the results of search queries in Korean. The data are normalized by the maximum value for the whole query period. They are then multiplied by 100.

url : <http://datalab.naver.com/ca/list.naver?seq=4559>

| Date | Search Query | |
| --- | --- | --- |
|  | # of AlphaGo in search query | # of Sedol Lee in search query |
| 2016-02-01 | 0.049940483 | 0.339611113 |
| 2016-02-02 | 0.034902937 | 0.202729869 |
| 2016-02-03 | 0.03051039 | 0.188800352 |
| 2016-02-04 | 0.058804721 | 0.388483139 |
| 2016-02-05 | 0.081558903 | 0.316342489 |
| 2016-02-06 | 0.029125353 | 0.18512011 |
| 2016-02-07 | 0.015512416 | 0.109536654 |
| 2016-02-08 | 0.016857881 | 0.156944497 |
| 2016-02-09 | 0.020894275 | 0.159556282 |
| 2016-02-10 | 0.036683699 | 0.256627601 |
| 2016-02-11 | 0.02176487 | 0.229678735 |
| 2016-02-12 | 0.01907394 | 0.178669794 |
| 2016-02-13 | 0.012307045 | 0.093549367 |
| 2016-02-14 | 0.012861059 | 0.109140929 |
| 2016-02-15 | 0.026434424 | 0.127146412 |
| 2016-02-16 | 0.039255911 | 0.214126746 |
| 2016-02-17 | 0.03320132 | 0.132053401 |
| 2016-02-18 | 0.027225873 | 0.108151616 |
| 2016-02-19 | 0.071744925 | 0.561771075 |
| 2016-02-20 | 0.025365966 | 0.119667211 |
| 2016-02-21 | 0.02129 | 0.219864757 |
| 2016-02-22 | 0.71566849 | 8.107532703 |
| 2016-02-23 | 0.555202041 | 5.650081044 |
| 2016-02-24 | 0.159397992 | 0.515471261 |
| 2016-02-25 | 0.111792286 | 0.278313325 |
| 2016-02-26 | 0.159437564 | 0.408111094 |
| 2016-02-27 | 0.027384163 | 0.047526561 |
| 2016-02-28 | 0.112267155 | 0.669289531 |
| 2016-02-29 | 0.058131989 | 0.344122377 |
| 2016-03-01 | 0.18701959 | 1.520731236 |
| 2016-03-02 | 0.230826337 | 3.662552711 |
| 2016-03-03 | 3.156420557 | 28.31209873 |
| 2016-03-04 | 1.319742557 | 27.10889716 |
| 2016-03-05 | 1.445068635 | 16.31146399 |
| 2016-03-06 | 0.406369905 | 1.619385455 |
| 2016-03-07 | 5.554711342 | 2.273439578 |
| 2016-03-08 | 33.0323054 | 37.63818714 |
| 2016-03-09 | 54.53643202 | 100 |
| 2016-03-10 | 32.57718219 | 70.35240886 |
| 2016-03-11 | 20.25138029 | 23.46185687 |
| 2016-03-12 | 20.10630754 | 36.03332479 |
| 2016-03-13 | 20.9785248 | 68.16488116 |
| 2016-03-14 | 11.70506781 | 41.46460953 |
| 2016-03-15 | 13.7255996 | 66.49610923 |
| 2016-03-16 | 3.771139624 | 14.88701263 |
| 2016-03-17 | 1.787766402 | 5.227011549 |
| 2016-03-18 | 1.26750687 | 2.611190784 |
| 2016-03-19 | 1.16343122 | 2.447875116 |
| 2016-03-20 | 1.003439641 | 1.495602705 |
| 2016-03-21 | 0.859277058 | 1.699440603 |
| 2016-03-22 | 0.622633565 | 0.998374362 |
| 2016-03-23 | 0.520892692 | 0.69358704 |
| 2016-03-24 | 0.490065722 | 0.605617394 |
| 2016-03-25 | 0.426591447 | 0.573761539 |
| 2016-03-26 | 0.582427915 | 0.641153491 |
| 2016-03-27 | 0.943724753 | 0.672217896 |
| 2016-03-28 | 1.013253619 | 0.528886335 |
| 2016-03-29 | 0.512186744 | 0.465609923 |
| 2016-03-30 | 0.482072079 | 16.93975642 |
| 2016-03-31 | 0.443686763 | 2.275616065 |
| 2016-04-01 | 0.408229812 | 0.411197748 |
| 2016-04-02 | 0.324177842 | 0.336405741 |
| 2016-04-03 | 0.412661931 | 0.368578176 |
| 2016-04-04 | 0.358882916 | 0.339215388 |
| 2016-04-05 | 0.287810723 | 0.3114355 |
| 2016-04-06 | 0.322357507 | 0.588482506 |
| 2016-04-07 | 0.273881207 | 0.311395928 |
| 2016-04-08 | 0.132330408 | 0.185555408 |
| 2016-04-09 | 0.308586281 | 0.317529664 |
| 2016-04-10 | 0.847998898 | 0.343251782 |
| 2016-04-11 | 0.289551913 | 0.255915296 |
| 2016-04-12 | 0.203837898 | 0.235614608 |
| 2016-04-13 | 0.191253846 | 0.181835594 |
| 2016-04-14 | 0.192203586 | 0.159714572 |
| 2016-04-15 | 0.163117806 | 0.173564943 |
| 2016-04-16 | 0.146418215 | 0.214245463 |
| 2016-04-17 | 0.167431207 | 0.264937824 |
| 2016-04-18 | 0.158606542 | 0.176335018 |
| 2016-04-19 | 0.153066393 | 0.255519571 |
| 2016-04-20 | 0.154253568 | 0.329520128 |
| 2016-04-21 | 0.14681394 | 0.255202991 |
| 2016-04-22 | 0.130391356 | 0.345705277 |
| 2016-04-23 | 0.128610594 | 0.295131634 |
| 2016-04-24 | 0.143450278 | 0.537196558 |
| 2016-04-25 | 0.105618977 | 0.261613735 |
| 2016-04-26 | 0.109457509 | 0.222357824 |
| 2016-04-27 | 0.143925148 | 0.426710165 |
| 2016-04-28 | 0.123505743 | 0.363354608 |
| 2016-04-29 | 0.111515278 | 0.325365017 |
| 2016-04-30 | 0.086822044 | 0.249108828 |
